# Highly stable active core microbiomes in Greenland cryoconite holes during the bare ice period

**DOI:** 10.1093/femsec/fiag074

**Published:** 2026-07-08

**Authors:** Helen K Feord, Athanasios Zervas, Katie Sipes, Ate H Jaarsma, Martyn Tranter, Liane G Benning, Alexandre M Anesio

**Affiliations:** Interface Geochemistry, GFZ Helmholtz Center for Geosciences, 14473 Potsdam, Germany; Department of Environmental Science, Aarhus University, 4000 Roskilde, Denmark; Department of Environmental Science, Aarhus University, 4000 Roskilde, Denmark; Department of Environmental Science, Aarhus University, 4000 Roskilde, Denmark; Netherlands Institute of Ecology (NIOO-KNAW), 6708 PB Wageningen, Netherlands; Department of Environmental Science, Aarhus University, 4000 Roskilde, Denmark; Interface Geochemistry, GFZ Helmholtz Center for Geosciences, 14473 Potsdam, Germany; Department of Earth Sciences, Freie Universität Berlin, 12249 Berlin, Germany; Department of Environmental Science, Aarhus University, 4000 Roskilde, Denmark

**Keywords:** cryoconite holes, Greenland Ice Sheet, melt season, metatranscriptomics

## Abstract

Cryoconite holes host diverse microbiomes on glaciers and ice sheets and are important habitats for supraglacial biogeochemical cycling. Despite reports of relative stability of cryoconite hole community composition on the Greenland Ice Sheet, it is not known how microbial function in cryoconite holes evolves under varying environmental conditions. Here, we address this knowledge gap by quantifying the active community members in five cryoconite holes on the Greenland Ice Sheet over a 3-week period during the 2022 melt season using TotalRNA. Active microbiomes were enriched in cyanobacterial sequences (25%–50%). Spatial variation between cryoconite holes had a greater impact than temporal variation on community composition quantified by rRNA SSUs, suggesting location-distinct and highly stable active microbiomes. In contrast, minor temporal variations in gene expression were identified in the mRNA data (∼1% of quantified transcripts), mostly due to cellular stress responses from washed-in glacier ice algae. Photosynthesis was the dominant active function across all surveyed cryoconite holes and time points, associated with both cyanobacterial taxa and washed-in chlorophyte snow algae. Overall, our data indicate that core cryoconite hole communities stably ensure their ecological roles, such as carbon fixation, regardless of variations in weather, highlighting their resilience and self-sufficiency.

## Teaser

Cryoconite holes on the Greenland Ice Sheet host active microbiomes that are highly stable (quantified via total RNA quantification) during the summer melt season.

## Introduction

Different supraglacial habitats exist, including bare ice and melting snow packs, however, cryoconite holes have long been regarded as the main hot-spots of microbial activity on glaciers and ice sheets (Anesio et al. 2009, 2017). Cryoconite holes are water-filled depressions on glacier and ice sheet surfaces filled with dark particulate matter (granules) formed from the interplay between minerals, microbes, and organic matter (Nordenskiöld 1875, Langford et al. 2010, Cook et al. 2016, Rozwalak et al. 2022). These holes form by the solar heating-in of these dark sediments/granules, reducing ice surface albedo, resulting in surface melting and sediment/granule accumulation below the glacier surface. Cryoconite granules provide a growth substrate for a very diverse microbial community (Takeuchi et al. 2010, Edwards et al. 2014, Vonnahme et al. 2016), with both aerobic (on granule surfaces) and anaerobic microhabitats (inside the granules; Poniecka et al. 2018, Segawa et al. 2020). Cyanobacterial taxa, including members of the *Phormidesmis, Tychonema* and *Scytonema* genera (e.g. Segawa et al. 2017, Millar et al. 2021, Zhu et al. 2025), are considered to be keystone taxa for cryoconite holes. Cyanobacteria are the most abundant primary producers in these habitats (Cameron et al. 2012, Jaarsma et al. 2023), thus ensuring the availability of organic carbon for ecosystem heterotrophs. Beyond carbon fixation, cyanobacteria also play a key ecosystem engineering role as filamentous micro-organisms, producing extracellular polymeric substances (EPS), entangling debris and contributing to the formation of granules (Langford et al. 2010, Anesio et al. 2017, Wejnerowski et al. 2023, Zhu et al. 2025). Overall, cyanobacterial taxa have been shown to be essential for microbiome stability in cryoconite holes (Zhu et al. 2025), thus making them a key player for our understanding of cryoconite hole ecosystem dynamics.

Other prokaryotic cryoconite hole community members include Pseudomonadota, Bacteroidota, and Actinobacteriota (e.g. Bourquin et al. 2022, Jaarsma et al. 2023, Ge et al. 2026). Eukaryotes also fulfil distinct ecosystem functions in cryoconite hole granules, including predators (protists belonging to Cercozoa and Ciliophora; Cameron et al. 2012, Millar et al. 2021, Jaarsma et al. 2023), meiofauna consumers (tardigrades and rotifers; Zawierucha et al. 2021), fungal decomposers (in particular basidiomycetes; Edwards et al. 2013, Jaarsma et al. 2023), and fungal parasites (chytrids; Jaarsma et al. 2023, Kobayashi et al. 2023). Cryoconite hole microbial communities are known to be key actors in biogeochemical cycles (Segawa et al. 2014, Musilova et al. 2017, Sanyal et al. 2018, 2020, Antony et al. 2024, Chen et al. 2025). For example, cyanobacteria and their associated microbiomes in cryoconite hole granules fix carbon and subsequently produce and transform different organic molecules (Murakami et al. 2022, Chen et al. 2025). Important nitrogen, sulphur, methane, and iron cycling have also been reported (Murakami et al. 2022, Antony et al. 2024, Chen et al. 2025). Overall, cryoconite holes have been described as self-sustaining habitats within the glacier ecosystem because of the diversity of their taxa and their various biogeochemical roles (Antony et al. 2024).

Cryoconite hole community compositions are known to vary spatially at a regional (Edwards et al. 2011, Cameron et al. 2016, Liu et al. 2017) and global scale (Cameron et al. 2012, Edwards et al. 2014, Darcy et al. 2018, Millar et al. 2021, Ge et al. 2026). Geographical variations in radiation (Zhang et al. 2023), nutrient inputs (e.g. nitrogen; Murakami et al. 2022), local bedrock/mineral composition (Rozwalak et al. 2022), or anthropogenic inputs (Ge et al. 2026) have all been shown to shape global cryoconite hole community differences. At local scales, significant intra-glacier similarity in cryoconite granule community composition has been reported for spatially separated cryoconite holes (Zhu et al. 2025), indicating location-stabilized cryoconite hole communities.

Cryoconite hole community composition is also known to vary temporally due to changes in environmental conditions, including stochastic weather events, extreme weather conditions such as storms, high precipitation, variable radiation, cloud cover (Chen et al. 2022, Zhang et al. 2023), and/or seasonal environmental changes in photoperiod and mean daily temperatures (Sanyal et al. 2024). Invariably, the prevailing environmental conditions impact on basic microbial requirements, such as oxygen availability, light, nutrient availability (including mineral dust), liquid water, or substrate availability (Chen et al. 2025), and thus all have the potential to affect cellular and ecosystem function. Furthermore, the physical development of cryoconite holes is also sensitive to weather conditions, developing more under sunny conditions and collapsing under low shortwave solar radiation linked to clouds, rain, and wind (Cook et al. 2016, Takeuchi et al. 2018), thus altering the habitat for cryoconite hole microbes (Takeuchi et al. 2025). Melt season duration, temperature, and radiation-controlled cryoconite hole depth (which contributes to variations in light availability for the sediments) have been shown to impact cryoconite hole community structure and metabolism (Cook et al. 2016, Gokul et al. 2016, Franzetti et al. 2017, Takeuchi et al. 2025). Stochastic weather events also impact cryoconite hole communities, such as the negative impact of wind and heavy rain on invertebrates reported in Svalbard (Zawierucha et al. 2019), and the decrease in Tibetan plateau cryoconite hole bacterial community diversity following a snowstorm (Chen et al. 2022). Temperate glacier cryoconite holes have been identified as unstable through the melt seasons (Franzetti et al. 2017, Pittino et al. 2018, Jaroměřská et al. 2025), yet cryoconite hole community composition on the Greenland Ice Sheet (GrIS) following snow melt have been identified as highly temporally stable through a melt season (Musilova et al. 2015, Stibal et al. 2015).

Cryoconite holes communities on the GrIS are known to be important contributors of organic carbon through the melt season (e.g. Musilova et al. 2017), and likely contribute to other important biogeochemical cycles such as N and P. Despite accumulated knowledge on GrIS cryoconite hole community composition (e.g. Musilova et al. 2015, Stibal et al. 2015, Jaarsma et al. 2023, Takeuchi et al. 2025), very little information is available regarding microbiome function in GrIS cryoconite holes, microbiome resilience to environmental change, and the stability of microbiome contributions to biogeochemical cycling on supraglacial surfaces. Here, this knowledge gap is addressed by measuring microbial activity in GrIS cryoconite holes via rRNA and mRNA quantification, using a TotalRNA sequencing approach (Trivedi et al. 2022, Perini et al. 2024). We hypothesized that the active cryoconite hole communities remain fairly stable through the melt season, but that their functional metabolic responses vary according to weather, e.g. with the development of lids and with seasonal and stochastic changes in light availability.

## Materials and methods

### Sampling on the Greenland Ice Sheet

**Figure 1.**
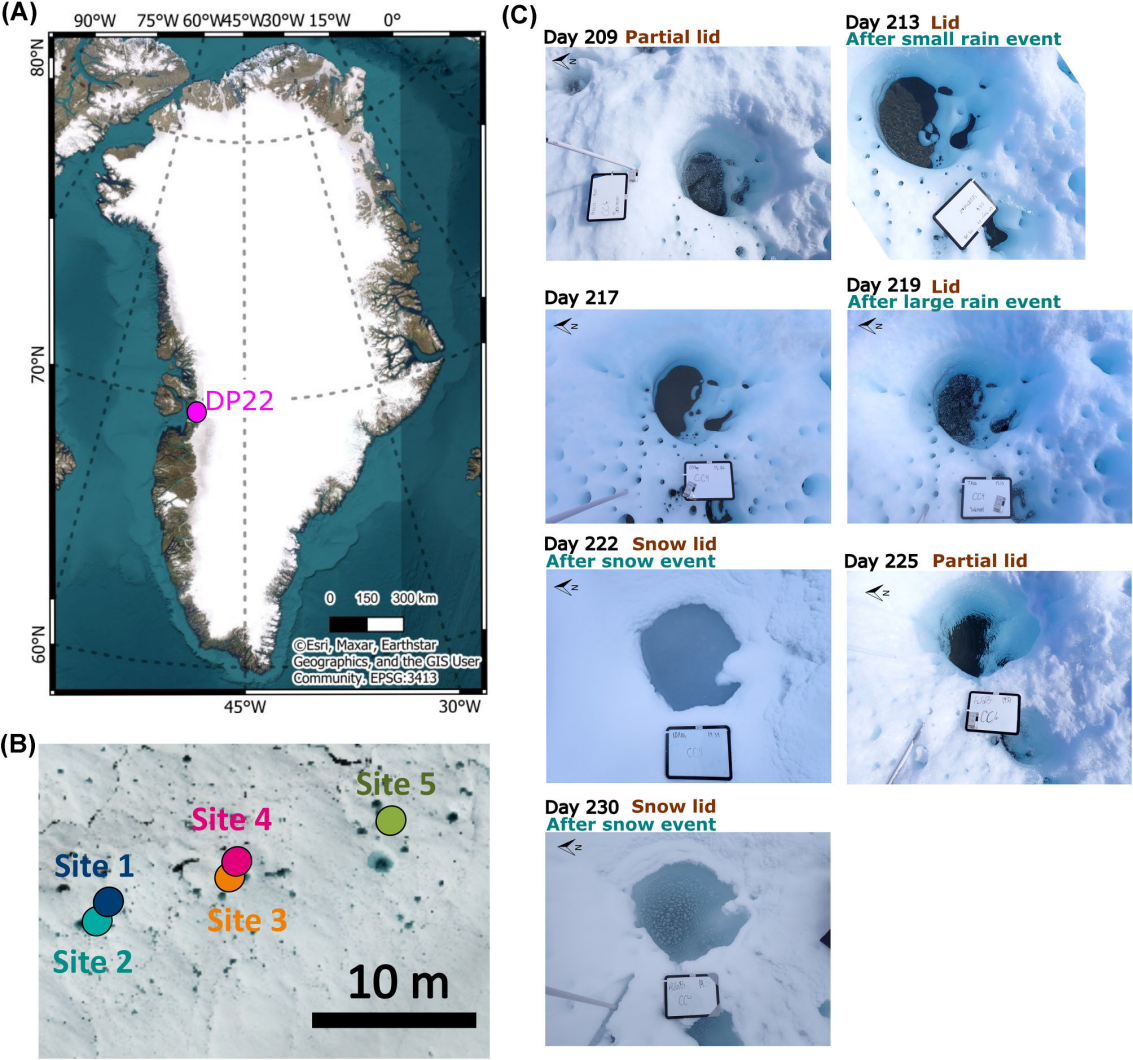
Cryoconite hole sampling location and description on the Greenland Ice Sheet. (A) Sampling was undertaken during the 2022 melt season (DP22) in W-Greenland near Ilulissat (Image credit: Shunan Feng). (B) Site map (Drone image credit: Shunan Feng) with five sampled cryoconite holes: sites 1 and 2 in dark / light blue, sites 3 and 4 in orange and pink, and site 5 in light green (image credit: Shunan Feng). (C) Pictures labeled with day of year (D209 to D230) show temporal cryoconite hole evolution (using site 4 as an example), associated weather conditions (green), and the presence or not of a lid during the sampling period (red). All cryoconite hole pictures through the melt season are available in SFig. 1. The white board in each picture can be used for scale (∼30 cm width).

The sampling strategy used in this study has previously been described by Jaarsma et al. (2025). In brief, sampling was undertaken during the DEEP PURPLE ice camp near Ilulissat (69.43 N, 49.86 W, 680 m asl) in the summer of 2022 (Fig. 1A). Temporal sampling of sediment material from five cryoconite holes of similar morphology and within 20 m of each other Fig. 1B and C, SFig. 1) was done regularly at 2 pm (representing solar noon) over 21 days from July 28^th^ to August 18^th^ (hereafter day of year 209–230; Fig. 1C). During the full 21-day sampling period, cryoconite holes varied slightly in size and morphology (Fig. 1, SFig. 1–2), mostly linked to a variable water level. Site 5 was the largest cryoconite hole surveyed. Any changes in cryoconite hole size during the sampling period did not follow a particular trend (SFig 1–2). During the sampling period, there was also evidence of merging between the studied cryoconite holes and smaller neighbouring cryoconite holes (SFig. 1–2). Cryoconite hole diameters were determined using site pictures (SFig. 1) and ImageJ v.1.54 g (Schneider et al. 2012).

**Figure 2.**
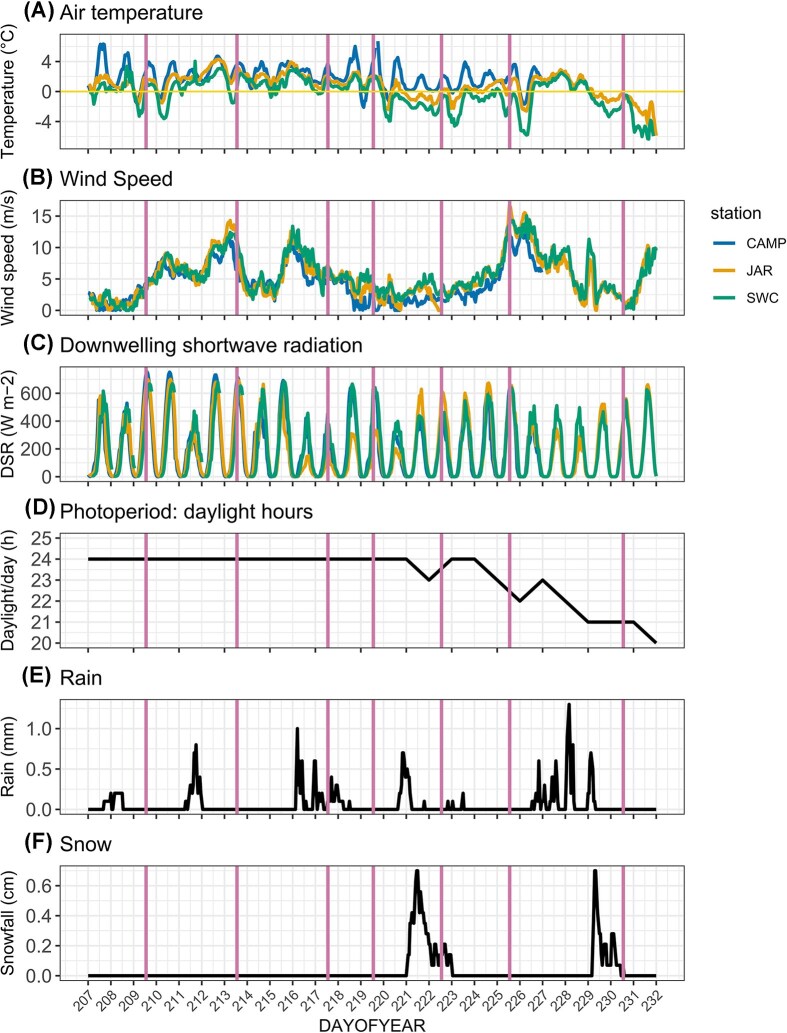
Weather conditions for the duration of the sampling season. Data originates from the DP22 camp weather station (“CAMP”), two nearby PROMICE weather station (“JAR” and “SWC”) and open-meteo.com. Data presented includes(A) air temperature (from CAMP and PROMICE weather stations), (B) wind speed (from CAMP and PROMICE weather stations), (C) downwelling shortwave radiation (from CAMP and PROMICE weather stations), and (D) photoperiod (calculated from the downwelling shortwave radiation from the JAR weather station, assuming that all values above 0 W.m^-2^ are daylight hours). For technical reasons the last 5 days of the CAMP weather station are missing, therefore we used data from open-meteo.com for our camp location for (E) rain and (F) snow events (snow events are also visible via the photographs in SFig. 1).

The 21-day sampling period matches the “late summer ice surface” described by Chandler et al. (2015; mid-July to mid-August): characterized by mostly positive air temperatures, with snow fully melted (except above crevasses and other depressions), varying ice surface darkness, and stable cryoconite holes (Fig. 2, SFig. 1). The ice surface was snow-free from the beginning of the sampling period. However, two important rain events occurred previous to two sampling days (D213 and D219) and two snowfall events occurred: on August 9^th^ (D221) with the snow remaining until August 13^th^ and thus on the ground during sampling day D222 and August 17^th^ (D229) with the snow remaining until August 19^th^ (D231) and thus on the ground during sampling day D230 (Figs. 1C, 2, SFig. 1). Relevant weather variables (wind, precipitation, air temperature, radiation, and photoperiod) are available in Fig. 2, and the full weather data set can be found in Jaarsma et al. (2025) for the camp weather station and via https://promice.org/ (Fausto et al. 2021) for the JAR and SWC weather stations that were, respectively, ∼10 and ∼30 km away from base camp. Rain and snow data for this location were also downloaded from open-meteo.com (Zippenfenig 2024).

Sediments/granules from the base of each of the cryoconite holes was collected with a polycarbonate aquarium pipette directly into 5 ml cryotubes, and stored and transported to the home laboratory in a liquid nitrogen-cooled cryoshipper, and subsequently stored in a −80°C freezer until extraction.

### RNA extractions and analysis

RNA was extracted as previously described by Jaarsma et al. (2025). Briefly, RNA and DNA were co-extracted in all samples using NucleoBond RNA Soil Mini and DNA Extraction kit (NucleoBond, Germany), using ∼0.5 g (wet weight) of cryoconite hole sediment. Nucleotide extraction was performed following manufacturer’s instructions with the addition of the 100 µl proprietary “Buffer OPT” (NucleoBond, Germany) which is recommended for mineraloid samples. After coextraction of DNA and RNA, concentrations were assessed with QuBit Fluorometic Quantification (ThermoFisher Scientific, USA). The RNA concentration and fragment size distribution were evaluated for fragment size on the Tapestation 4150 RNA (Agilent Technologies, USA). RapidOut DNA Removal kit (Thermo Scientific) was used to remove residual DNA before RNA library preparation. NEBNext Ultra II Directional RNA Library Prep with Sample Purification Beads (E7765, New England Biolabs, USA) was used for RNA library preparation. Insert size distribution and the presence of adapter-primer dimers were checked using a TapeStation 4150. The libraries were subsequently pooled (equimolarly) sequenced on a NextSeq 500 (Illumina) using the 300 cycles (151 bp paired-end sequencing) chemistry. Information regarding RNA yields and sequencing depth is available in Supplementary table 1. Due to a sample storage issue in non-frozen conditions, three samples (Site 1 day 217 and 222, and Site 5 day 222) were excluded for further analysis.

### rRNA and mRNA assembly and annotation

The analysis of raw paired-end Illumina fastq reads was undertaken using the automated TotalRNA analysis pipeline developed at Aarhus University (Department of Environmental Science, available on GitHub; Jiménez 2023, Scheel et al. 2023). Briefly, TrimGalore (https://github.com/FelixKrueger/TrimGalore) was used for read trimming and quality control, including the removal of a adapters and short reads. rRNA sequences were subsequently sorted into small subunit rRNA, large subunit (LSU) rRNA, and non-rRNA sequences using SILVA 138.1 SSU Ref NR 99 with the SortMeRNA software (Kopylova et al. 2012). MetaRib (Xue et al. 2020) was used to reconstruct full-length ribosomal genes from the SSU reads, which was evaluated with Quast (Gurevich et al. 2013). Assembled rRNA contigs were taxonomically classified using CREST4 (Lanzén et al. 2012) using the SILVA database v.138. BWA (Li and Durbin 2009) and Samtools (Danecek et al. 2021) were used to estimate rRNA abundances. Phylogenetic analysis was undertaken to assign cyanobacterial sequences to a higher taxonomical resolution. Homologous sequences to the identified cyanobacterial sequences were searched via BLAST on NCBI (https://www.ncbi.nlm.nih.gov/). All sequences were subsequently aligned with Clustal-Omega (v1.2.4; Sievers et al. 2011), trimmed with TrimAL (with -automated; Capella-Gutiérrez et al. 2009), and maximum likelihood (ML) trees were built with IQ-TREE (v2.4.0, -m MFP, -B 1000; Minh et al. 2020). The ML tree was visualized and annotated with iTOL (Letunic and Bork 2024). Cyanobacteria classification followed the updated cyanobacterial phylogeny established by Strunecký and colleagues (Strunecký et al. 2023).

mRNA reads were also retrieved using SortMeRNA, from the sequence unassigned to rRNAs and mRNA contigs, assembled using Trinity, and BWA and Samtools were used to map reads to mRNA contigs and estimate mRNA abundances. Transcript Open Reading Frames (ORFs) were predicted using Transdecoder (v.5.7.0). When multiple ORFs were predicted per contig, only the longest one was retained for each contig. ORFs taxonomy was assigned using DIAMOND blastp (v. 0.8.22) against the NCBI nr database (Buchfink et al. 2015), and the Lowest Common Ancestor (LCA) of each sequence was assigned with MEGAN v. 6.24.23 (Huson et al. 2016). ORFs were functionally annotated with via GhostKOALA (Kanehisa et al. 2016) for KEGG pathways and InterProScan (v. 5.65–97; Jones et al. 2014) for Gene Ontology (GO) terms.

### Statistical analysis

Data analysis was undertaken using R (R Core Team 2023). Data analysis and plotting was partially undertaken with the phyloENVS (v. 0.1.0) R package (https://github.com/AU-ENVS-Bioinformatics/phyloENVS/tree/main). The R packages phyloseq (v. 1.44.0; McMurdie and Holmes 2013) and vegan (v. 2.7–2 ; Dixon 2003) were used for statistical analysis. For NMDS and PERMANOVAs, mRNA data was normalised with DESeq2 (v. 1.40.2; Love et al. 2014), using the vst() function (blind = TRUE). PERMANOVA tests were performed with vegan using Bray–Curtis distance metric and 999 permutations. To identify transcripts which varied across sites and time, likelihood ratio tests (LRT, padj <0.05) were undertaken with DESeq2, followed by cluster analysis with Mfuzz (v. 2.60.0) to bin significantly variable transcripts by expression pattern (Kumar and Futschik 2007). GO enrichment for DEGs for each identified cluster was undertaken with topGO (v. 2.25.0; Adrian Alexa 2017).

## Results

### Spatially distinct but taxonomically stable active cryoconite hole communities show functional variation through the melt season

**Figure 3.**
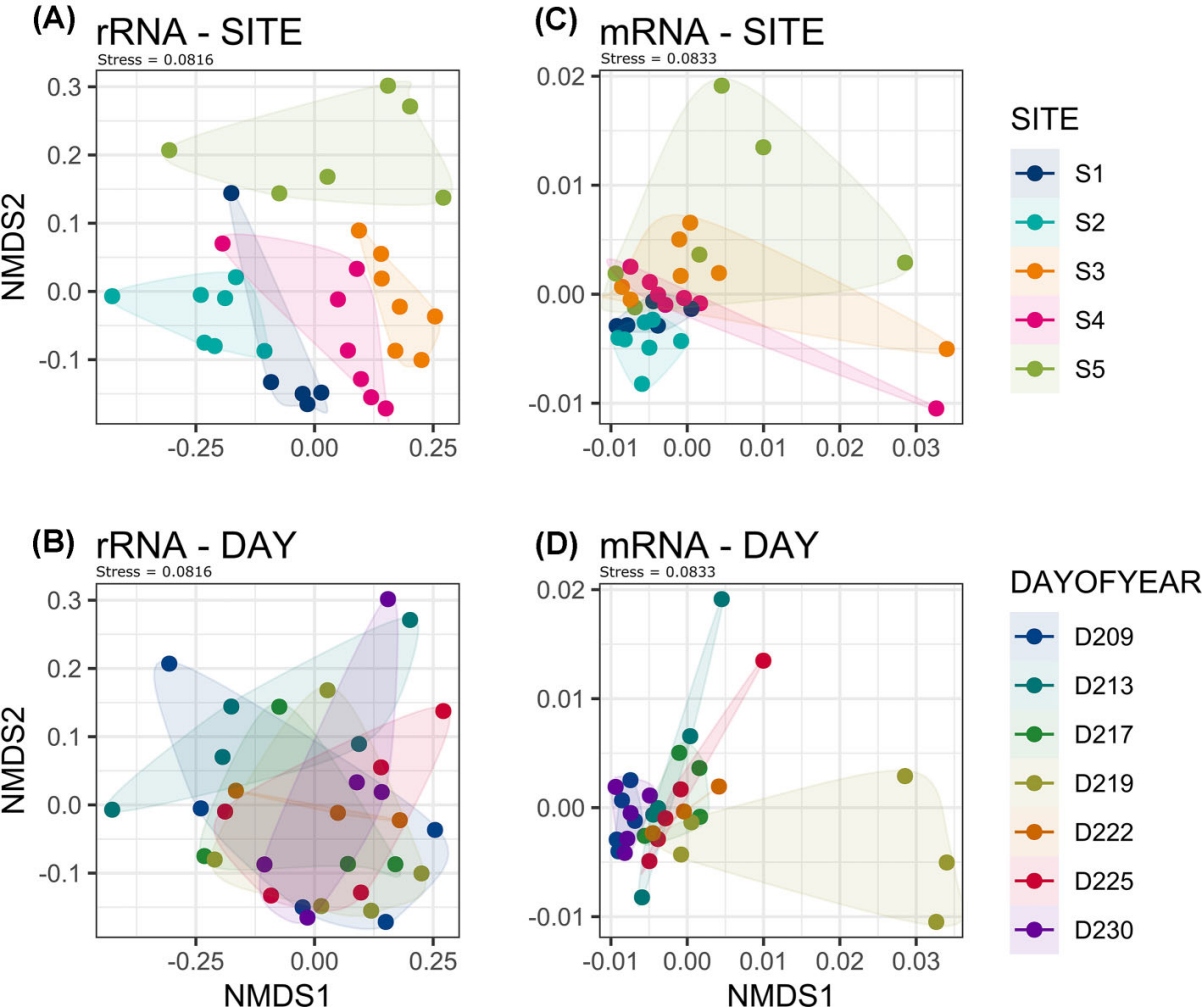
NMDS plots of (A, B) rRNA and (C, D) mRNA datasets for all five cryoconite hole sites and all seven time points. Plots are color-coded either by site (A, C) or day (B, D). The rRNA NMDS plots were built using relative abundance data, and the mRNA NMDS plots using DESeq2 normalized data.

The differences between samples were visualised by NMDS for both rRNA (using relative abundance, clustered by site Fig. 3A, and by sampling day Fig. 3B) and mRNA (using DESeq2 transformed data, clustered by site Fig. 3C, and by sampling day Fig. 3D). Clear separation of samples by site was found (Fig. 3A and C), a separation also supported by PERMANOVAs (rRNA: PERMANOVA, R^2^ = 0.53. F-Model = 7.72, *P =* 0.001, mRNA: PERMANOVA, R^2^ = 0.18 F-Model = 1.49, *P =* 0.001). However, we also identified sample separation by sampling day for the mRNA data (PERMANOVA, R^2^ = 0.27 F-Model = 1.51, *P =* 0.001), a separation not observed based on the rRNA data (PERMANOVA, R^2^ = 0.15. F-Model = 0.71, *P =* 0.884) (Fig. 3B and D). In particular, mRNA samples from three sites (sites 3-5) formed a separate group on day 219 (bottom-left of mRNA NMDS plots, Fig. 3C and D). This indicates that the five sites contained spatially distinct microbial community compositions, but that over the 21 sampling days, with varying weather conditions including rain and snow events, and a variation in photoperiods (Fig. 2), no seasonal succession in the cryoconite hole microbiomes was observed (Fig. 3A-C). In contrast, a temporal variation in community function was identified (Fig. 3D).

### Active cryoconite hole communities are dominated by Cyanobacteria

**Figure 4.**
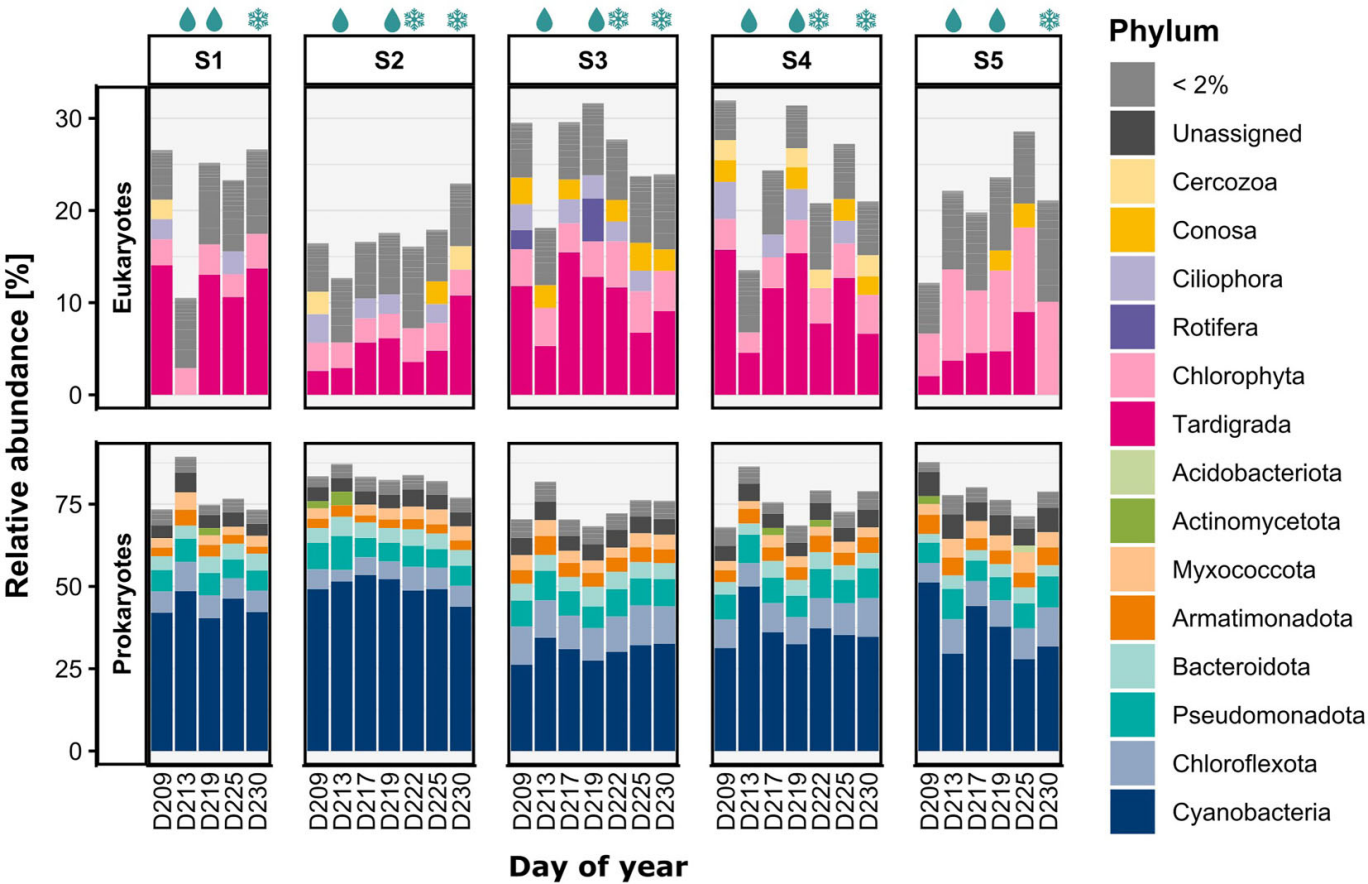
Relative abundance of active cryoconite hole community members based on taxonomic annotation of rRNA SSUs at phylum level. Relative abundance is plotted for each cryoconite hole (site 1–5) across the different sampling points (D209-230). Community members are divided by eukaryotes and prokaryotes, note the different *y*-axis scale. All phyla under 2% are not shown individually (“<2%”, shown in grey). Important rain and snow events are marked by a liquid drop and an ice crystal. Please note that this signifies that the sample was taken after the marked weather event, not during.

The taxonomy of the active community was assessed by taxonomic classification of rRNA sequences using SILVA and the taxonomic classification of mRNA sequencing using the LCA (see methods). For the rRNA data, we quantified 535 rRNA SSUs across all five cryoconite hole sites (Supplementary Table 2), with ∼75% of the active cryoconite hole community sequences being made up of prokaryotes (Fig. 4). The main prokaryote sequences were cyanobacterial, making up ∼40%–50% of the total community in holes 1 and 2, but only ∼25%–30% of the community in holes 3–5 (Fig. 4).

**Figure 5.**
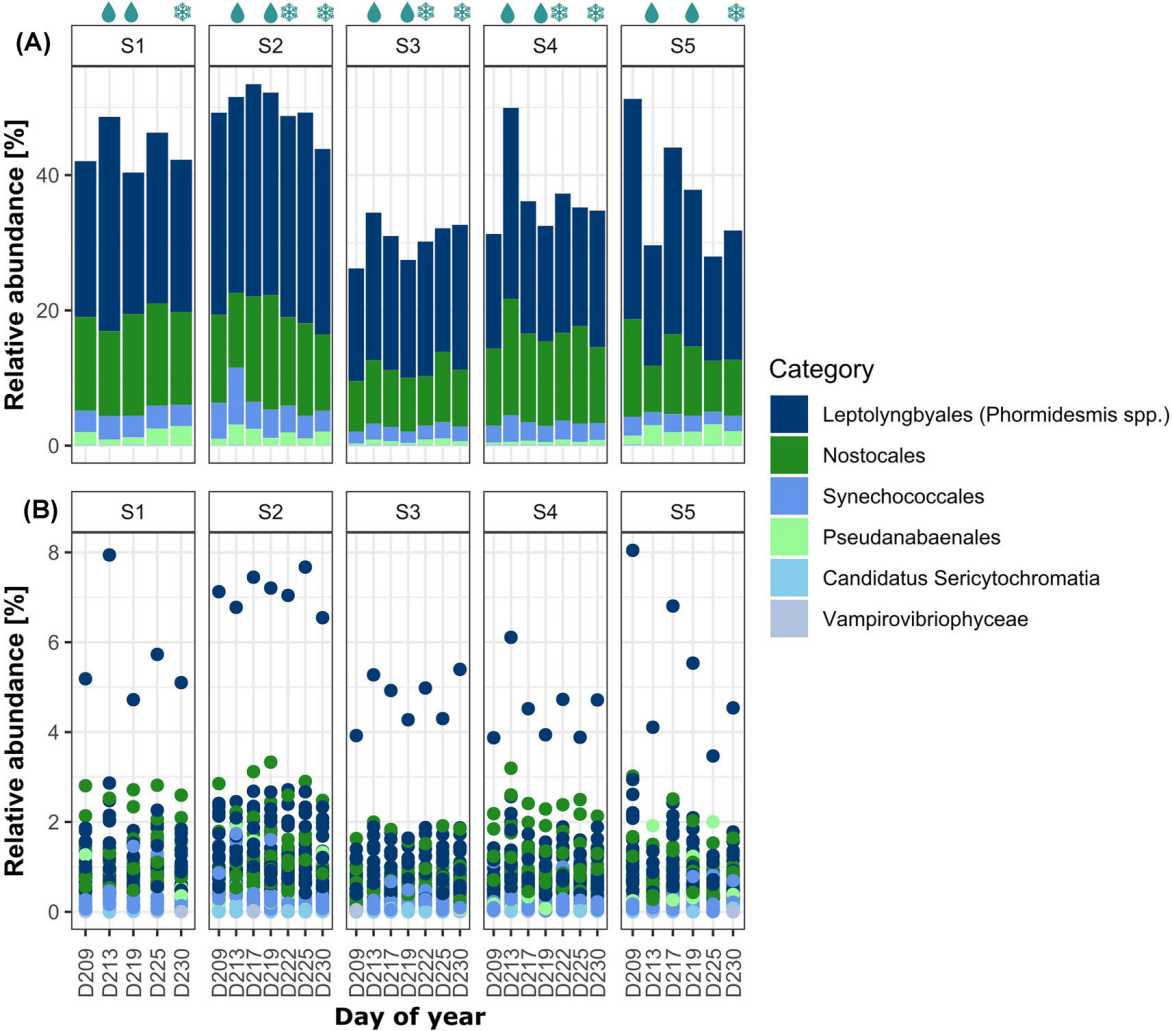
Relative abundance of active cyanobacterial cryoconite hole community members. Cyanobacterial sequences were taxonomically classified (at order or family level) via phylogenetic analysis using publicly available rRNA SSU sequences (SFig. 3). Relative abundance is plotted for each cryoconite hole (sites 1–5) across the different sampling points (D209-230), with (A) the relative abundance of the different cyanobacterial taxa plotted in relation to the total population and (B) the contribution of each cyanobacterial contig. Important rain and snow events are marked by a liquid drop and an ice crystal. Please note that this signifies that the sample was taken after the marked weather event, not during.

All cyanobacterial sequences were extracted and a more fine-tuned order and family-level taxonomic assignment was undertaken with phylogenetic analysis (Fig. 5, SFig. 3). The largest cyanobacterial fraction belonged to Leptolyngbyales (∼10%–30%), with all sequences clustering with *Phormidesmis priestleyi* 16S sequences (SFig. 3). Nostocales represented ∼6%–15% of the total community relative abundance, Synechococcales ∼1%–8%, and Pseudanabeanaceae ∼0.1%–2% (three low abundance sequences were annotated as Candidatus Sericytochromatia and two as Vampirovibriophyceae; (Fig. 5, SFig. 3). Notably, our GrIS cryoconite hole Nostocales group formed a separate cluster from the other closely related Nostocales genera on the phylogenetic tree (SFig. 3). One cyanobacterial Leptolyngbyales contig (LXYR01000057.208.1678_2, Supplementary Table 2), represented between 4% and 8% of the total rRNA sequences of the active community (Fig. 5B, visible as single dark blue data points above the remainder of the cyanobacterial taxa). The relative abundance for this single contig was markedly above all other cyanobacterial sequences (Fig. 5B), and was assigned to species level with SILVA (Supplementary table 2) as *Phormidesmis priestleyi* (Chrismas et al. 2016).

The other dominant active prokaryotic sequences belonged to Chloroflexota (∼5%–10%, mostly of the Anaerolineae class), Pseudomonadota (∼5%–7%, mostly of the Alphaproteobacteria class), Bacteroidota (∼4%), Armatimonadota (∼2%–4%, mostly of the Armatimonadia class), and Myxococcota (∼2%–4%, mostly of the Polyangia class; Fig. 4, SFig. 4). The dominant active eukaryotic sequences in the cryoconite hole samples belonged to tardigrades (∼2%–15% of total community, Fig. 4) and chlorophytes, which contributed between 2.5% and 10% of the total active community, with a particularly large fraction in site 5 (Fig. 4). The three other heterotrophic eukaryotic phyla that were ubiquitous across the cryoconite hole sites were Cercozoa (SAR, ∼1%–2% of total community), Ciliophora (SAR, ∼2%–4% of total community), and Conosa (Amoebozoa, ∼2% of total community). Microbial diversity was assessed by calculating alpha diversity with Shannon and Inverse Simpson measures (SFig. 5). For alpha diversity measures, Shannon diversity was ∼5, and Inverse Simpson diversity between 70 and 90 across all sites (SFig. 5A). Furthermore, we found lower alpha diversity measures for the eukaryotic fraction (SFig. 5B) of the community compared to the prokaryotic one (SFig. 5C). Regarding the taxonomic classification of the mRNA data, excluding the important proportion of contigs with no ORFs, we found similar patterns between mRNA and rRNA data: a large majority of prokaryotic counts, higher relative abundance of cyanobacteria mRNA in sites 1–2 compared to sites 3–5, and an enrichment of chlorophyte snow algae mRNA in site 5, and a higher relative abundance of Chloroflexi counts in sites 3–5 (SFig. 6). This observation was further corroborated by the taxonomic analysis of expression clusters of differentially abundant transcripts between sites (SFig. 7). Cluster analysis also revealed correlative expression patterns of chlorophyte snow algae mRNA and viral mRNA, with both in high abundance in site 5 (SFig. 6B, SFig. 7).

### Temporal variation in streptophyte mRNA is the main transient disturbance to cryoconite hole community function

**Figure 6.**
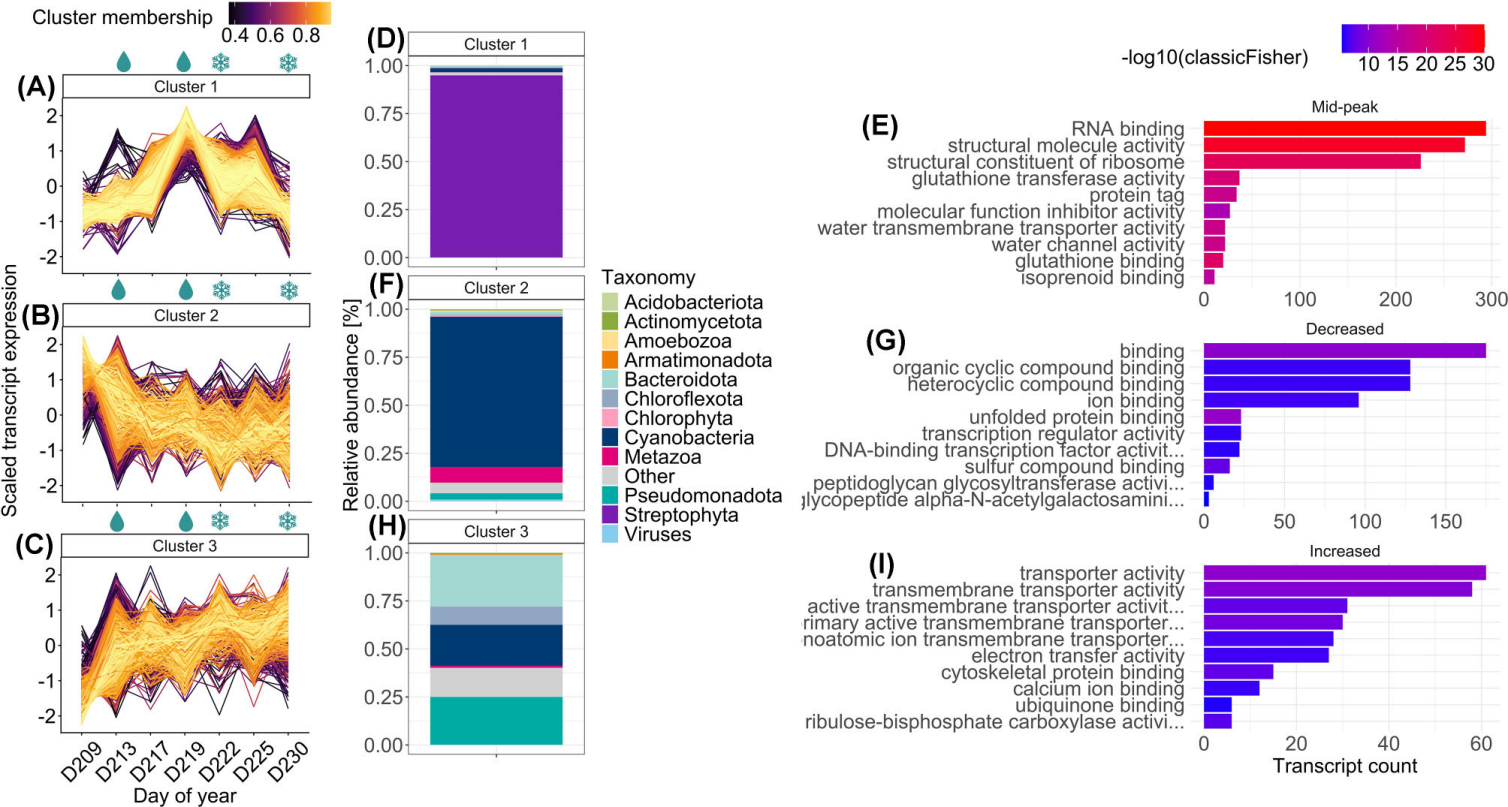
Expression clusters of temporally varying transcripts. The expression trends of the three clusters (A–C) are plotted by using scaled transcript expression and are color coded by cluster membership, with light colors indicating high cluster membership and darker colors low cluster membership. Important rain and snow events are marked by a liquid drop and an ice crystal. Please note that this signifies that the sample was taken after the marked weather event, not during. The taxonomy of the individual cluster transcripts (D, F, H) is plotted as the mean relative abundance of these transcript counts for the sample days with the highest expression (day 219 for cluster 1, day 209 for cluster 2, and day 230 for cluster 3), as well as the GO enrichments (D, G, I) for each cluster (with the number of transcripts with each GO term plotted on the *x*-axis).

We extracted all transcripts showing significant variability over time using DESeq2, allowing us to identify the transcripts responsible for driving sample separation by site (Fig. 3D). In total 4263 (3386 with an ORF, and 877 without) transcripts were identified as significantly variable, which consisted of ∼1.3% of all quantified transcripts (319 079 in total). Further grouping of the differentially regulated transcripts by temporal expression pattern produced three distinct clusters (Fig. 6A–C), of which cluster 1 contained the majority of transcripts. Cluster 1 (n = 3335, ∼1% of total transcripts) showed transcripts with a peak in expression at day 219 (Fig. 6A), a time point following a long heavy rain event (Fig. 2). Cluster 2 (n = 474, 0.15% of total transcripts), showed a lowering of transcript abundance through the sampling period (Fig. 6B). Finally, cluster 3 (n = 454, ∼0.14 of total community) showed an increase of transcript abundance through the sampling period (Fig. 6C). The similarities and dissimilarities seen between days in Fig. 3D largely match the trends in cluster 1 (for example, Days 209 and 230 are the most similar in Fig. 3D and are have the lowest abundances for transcripts in cluster 1), indicating that cluster 1 transcripts drive the NMDS separation in Fig. 3D.

Cluster 1 mainly consisted of transcripts assigned as streptophyte mRNA (Fig. 6D), enriched in GO terms linked to translation (RNA binding, structural constituent of a ribosome) and oxidative stress (Glutathione transferase activity, Fig. 6E). KEGG annotations of individual transcripts corroborated this finding, indicating the most abundant annotations of transcripts in cluster 1 included oxidative stress enzymes (glutathione S-transferases, peroxidases, etc), molecular chaperones (such as heat shock proteins), and proteins involved in biotic stress response (Chitinases and Pathogenesis-related proteins, SFig. 8). Streptophyte mRNA in cryoconite holes very likely belongs to glacier ice algae (Feord et al. 2025a) who thrive on the glacier ice surfaces surrounding the cryoconite holes. The increase in streptophyte transcripts in the mid-time course time points, in particular for sites 3–5 on day 219, in the relative abundance of mRNA taxonomy (SFig. 6B), was not mirrored in the amplitude for the rRNA data (SFig. 9). Cluster 2 (decreasing during the time series) was dominated by cyanobacterial transcripts (Fig. 6F), enriched in GO terms linked to transport (e.g. “transporter activity”) and energy production/photosynthesis (“electron transfer activity”, “ribulose-bisphosphate carboxylase activity”; Fig. 6G). Finally, cluster 3 (increasing during the time series) was made up of transcripts from different taxonomic groups, in particular Cyanobacteria, Proteobacteria, Bacteroidetes (Fig. 6H), and enriched in GO terms linked to compound binding (e.g. “ion binding”), transcription (e.g. transcription regulator activity), and bacterial cell wall growth (e.g. peptidoglycan glycosyltransferase activity; Fig. 6I).

### Photosynthesis is the most abundant active metabolism in cryoconite holes

**Figure 7.**
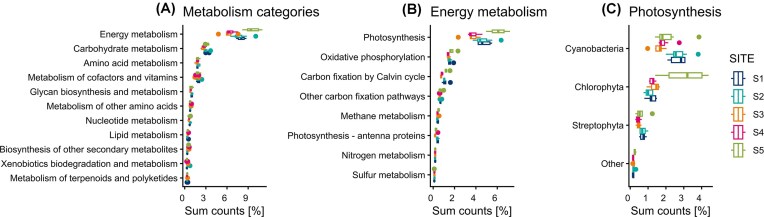
Sum of percentage counts of active cryoconite hole function based on functional annotation of mRNA with KEGG. (A) all KEGG categories belonging to metabolism and belonging to (B) energy metabolism. Sum of percentage counts is plotted for each cryoconite hole (sites S1–S5), using data points for all time points. Relative abundance of (C) photosynthesis transcripts (KEGG annotation) based on taxonomic annotation of mRNA using MEGAN at phyla level.

Approximately 98.7% of transcripts did not show variation across time. To assess the key metabolic groups that are stable through the melt season, we plotted the relative count contribution (sum percentage counts) of the transcripts to different metabolic pathways (Fig. 7). Across KEGG categories, the most abundant category was “Energy metabolism” (∼6%–9% sum percentage counts), followed by “Carbohydrate metabolism” and “Amino acid metabolism” (Fig. 7A). A closer look at the pathways in the abundant “Energy metabolism” KEGG category indicated that this functional group was mostly enriched in photosynthesis trancripts (∼4%–6% of sum percentage counts), making photosynthesis the most abundant metabolism function in our annotated mRNA data. In particular, site 5 had a markedly higher relative abundance of photosynthesis transcripts compared to the other sites (Fig. 7B). The taxonomic contribution of these photosynthesis transcripts varied between sites, with a relatively large fraction (∼1%–3%) of transcripts annotated as belonging to chlorophyte snow algae transcripts, in particular for site 5 (Fig. 7C). The other enriched pathways (in addition to the photosynthesis associated pathways “Photosynthesis—antenna proteins” and “Carbon fixation in photosynthetic organisms”) in the “Energy Metabolism” category were “Oxidative phosphorylation” (∼1%–2% total relative abundance) and “Methane Metabolism” (∼0.7%–1% of total relative abundance). Nitrogen and sulfur cycling transcripts were also represented, albeit at a low abundance (Fig. 7B).

## Discussion

This multi-site multi-day TotalRNA dataset captures the evolution of five active cryoconite hole microbial communities (located within ∼20 m of each other) over a short timescale (∼three weeks) during the 2022 melt season on the GrIS. The active cryoconite hole community composition (assessed using rRNA SSUs) was spatially distinct between holes, with no identified seasonal succession of community members (Figs. 3–5). rRNA sequences from the cyanobacteria phylum were the most abundant across all sites, of which the most abundant genus was *Phormidesmis* (Figs. 4 and 5). This matches previous analysis identifying *Phormidesmis* biosynthetic gene clusters as highly abundant in these samples (Jaarsma et al. 2025). The high relative abundance of cyanobacterial rRNA sequences was also associated with the most abundant metabolic function, photosynthesis, within the mRNA data, along with important contributions from chlorophyte snow algae taxa. A very small proportion of transcripts varied across time points, largely due to changes in streptophyte glacier ice algae stress response genes, however seasonal changes, such the decrease in daily light availability, appeared to have minimal impact on community function (Figs. 2 and 6).

The relative abundance of the active cryoconite hole community members significantly varied between the surveyed holes (Figs. 3 and 4), indicating consistent cryoconite hole site-differences existing at a local level. It must be noted however, that because these taxon profiles are reported as relative abundances, they should be interpreted as compositional data; therefore, these observed differences between sites reflect relative shifts within samples rather than absolute changes, and our result should be interpreted with this constraint in mind. This site-to-site variation result is consistent with previously reported intra-ice cap cryoconite metabolomes in Svalbard which highlighted consistent metabolic spatial variation of community function (Gokul et al. 2023), as well as cross-Greenland cryoconite hole variability in bacterial community composition (Cameron et al. 2016). Furthermore, our data is consistent with the cryoconite hole island biogeography hypothesis (Darcy et al. 2018). Indeed, with little community variation across timepoints (Fig. 3), we were able to quantify stable active communities, and assert that, despite the surveyed holes not having permanent lids (Darcy et al. 2018) neither hydrological (wash-in from melting snow or ice or from merging with smaller neighbouring cryoconite holes) nor aeolian input had a major effect on active community composition structure during our sampling period (Figs. 2, 4, SFig. 6). We offer three explanations for this temporally stable variation between cryoconite holes: 1) multi-season-induced community change, 2) proximity to algae-rich melting snow packs earlier in the melt season, and 3) variation in cryoconite hole size. For 1), as growth is slow in the cold, multi-season community turnover would amplify the impact of both stochastic and possibly deterministic processes on community composition over a longer period. Cryoconites are dynamic habitats, that can form and collapse due to variations in environmental conditions, such as the weather (Takeuchi et al. 2018). However, certain cryoconite types, the ice-lidded cryoconite holes in Antarctica, are known to persist and develop over multiple seasons (Bagshaw et al. 2007). A multi-season community turnover hypothesis concurs, for example, with the knowledge that inland GrIS cryoconite holes accumulate more organic carbon than at the margin (Stibal et al. 2010), suggesting stability over a longer period of time. Indeed, developed cryoconite holes located inland on the GrIS are less likely to collapse than holes at the margin due to flatter slopes, ultimately protecting the microbial communities from physical disturbances (Cook et al. 2016, Takeuchi et al. 2025), and thus more likely to persevere from year to year. Our second explanation (2) of temporally stable community variation, is linked to the abundance of chlorophyte snow algae, as we find that the relative abundance of chlorophyte snow algae varies between the surveyed cryoconite holes (Fig. 4). We can hypothesize that it is possible that this variation in community composition is, in part, determined by the proximity to melting algae-rich snow packs. Indeed, closer proximity could lead to an enrichment of chlorophyte snow alga (and their associated microbiomes) in cryoconite holes. Our third explanation (3), relates to variations in cryoconite sizes (both depth and diameter). Site 5 has a much bigger diameter than the other cryoconite holes (SFig. 1–2), a fact that could explain its clear separation from the other cryoconite holes in an NMDS plot of our rRNA dataset (Fig. 3A).

The cryoconite hole community members (Figs. 4 and 5), quantified with TotalRNA, are generally representative of previously described Greenland cryoconite hole microbial communities (Musilova et al. 2015, Stibal et al. 2015, Jaarsma et al. 2023, Takeuchi et al. 2025), and other cryoconite hole communities globally (Edwards et al. 2011, Franzetti et al. 2017, Liu et al. 2017, Lutz et al. 2019, Millar et al. 2021, Ge et al. 2026). Alpha diversity largely matches what has been previously published for cryoconite hole communities using amplicon sequencing and metagenomes for Shannon (∼5) and InvSimpson (∼80; Jaarsma et al. 2023, Ge et al. 2026). However, for cyanobacterial relative abundance, amplicon and metagenome sequencing of cryoconite holes in Greenland have reported quite a variation, ranging from ∼3% of total metagenome in south Greenland (Jaarsma et al. 2023), between 15% and 30% bacterial amplicon sequencing in west Greenland (Musilova et al. 2015, Stibal et al. 2015), and over 40% of bacterial DNA sequencing in north Greenland (Uetake et al. 2016). These differences could be biological (true differences between cryoconite holes at different sites on the GrIS) or technical due to varying sequencing approaches (different primer pairs, amplicon sequencing vs. metagenomics, improved taxonomic annotations). SSU copy number/cell ratio vary between taxa, even between species (Ruvindy et al. 2023), making direct comparisons difficult between eukaryotic and prokaryotic taxa abundance SSU copy numbers. However, given the smaller cellular size (thus a likely lower SSU copy number/cell) for cyanobacteria compared to many eukaryotes and the fact that our rRNA data shows a clear enrichment in cyanobacterial sequences, our data does provide good evidence that cyanobacteria are an important fraction of the active taxa in our surveyed cryoconite holes (Fig. 4). By focusing on the active community with RNA- rather than DNA-abundance, we document the importance of cyanobacteria as active cryoconite hole microbial communities on the GrIS. A consequence of the high cyanobacterial sequence relative abundance is the clear enrichment of photosynthesis as the most abundant expressed metabolic group (Fig. 7). Indeed, our TotalRNA data validates the important role of cyanobacteria as active primary producers in cryoconite holes (Gokul et al. 2023) and as the likely drivers of organic carbon availability in inland GrIS cryoconite holes (Stibal et al. 2010).

Our data also indicates that chlorophyte snow algae, known to have active photosystems in snow packs (Hamilton and Havig 2023, Feord et al. 2025b), may also be contributing to carbon fixation in cryoconite holes. Indeed, we observed an important presence of active chlorophyte snow algae in all sites, as well as chlorophyte snow algae photosynthesis transcripts (in particular site 5; Figs. 4 and 7C). Such a result is an important indication of the metabolic activity and ecological function of chlorophyte snow algae in these cryoconite hole habitats, despite the hypnozygote life stage that is often reported for these taxa on snow fields and bare ice on ice sheets and glaciers. However, the other main eukaryotic algal group, streptophyte glacier ice algae, typically very abundant on bare ice surfaces surrounding cryoconite holes, was very low in abundance in our rRNA data (Fig. 4, SFig. 9; Anesio et al. 2017). We can hypothesise that the difference between these two eukaryotic algal taxa in cryoconite holes could be attributed to the thick cell walls in snow algae (Ezzedine et al. 2023). Indeed, this cellular characteristic could provide snow algae with better protection from grazing and parasites compared to the thin cell walls of glacier ice algae. The grazing meiofauna (tardigrades) or algae-infecting chytrids present (Fig. 3), might actively be degrading the glacier ice algae that are washed into the cryoconite holes from the surrounding melting ice (Vonnahme et al. 2016, Kobayashi et al. 2023, Perini et al. 2026). Indeed, previous work has highlighted cryoconites as hotspots for chytrid infection of glacier ice algae (Kobayashi et al. 2023). In contrast, with this hypothesis, the chlorophyte snow algae and the cyanobacteria protected within EPS-mineral cryoconite hole granules would survive.

Indeed, our mRNA data shows an indication of a putative dynamic stress responses for the glacier ice algae in cryoconite holes, which cluster analysis indicating streptophyte stress response-related transcripts (Fig. 5, SFig. 9) with a peak of expression on day 219 (in particular for sites 3–5) and troughs on days 209 and 230. The response to abiotic stress, exemplified by the well-studied molecular chaperones heat shock response proteins seen in our data (SFig. 9), can be associated with a strong and transient induction of stress-protective genes (Pessa et al. 2024, Pratx et al. 2024). Indeed, the upregulation of translation proteins (Fig. 6), including heat shock proteins, provides the cells with the necessary machinery to produce and fold the new proteins required to protect the cells from incoming stress. This putative stress response could be due to grazing from tardigrades, as mentioned above, but could also be associated with abiotic stress. Indeed, cryoconite holes are a very different habitat to the bare ice surface that glacier ice algae normally bloom on (for example, full submersion in water and lowered oxygen availability), and could thus be considered to be stressful for the glacier ice algae. It is possible, that the increase in glacier ice algae mRNA at day 219 is linked to the heavy rain event that took place two days before (Fig. 2), as glacier ice algae will invariably be washed into the depression in cryoconite holes during melting and precipitation events.

A very small percentage of non-streptophyte transcripts were found to either increase or decrease in abundance throughout the three-week sampling time period (Fig. 6B and C), suggesting a response to seasonal change, for example with the reduction of daily light availability. The reduction of transport and energy production-related transcripts (mostly cyanobacterial) is consistent with lower light availability for photosynthesis and slower metabolism due to colder temperatures (Fig. 6B, F, and G). The increase in multi-taxa transcripts linked to organic compound binding and cell wall growth could suggest an increase in heterotrophic processes through the melt season (Fig. 6C, H, and I). However, in the case of both clusters (cluster 2 and 3), the number of differentially regulated transcripts is low (<0.5% of total transcript number), and thus is not representative of the great majority of quantified transcripts. We can therefore postulate that the gene expression underlying core biogeochemical cycling (carbon, nitrogen, sulfur, etc; Fig. 7) is predominantly stable under varying weather conditions (including the temporary development of cryoconite hole lids).

Our results clearly document cryoconite hole microbial community self-sufficiency. Cryoconite hole self-sufficiency (Antony et al. 2024) and a lack of response to non-extreme weather events (for example rain or snow events that do not impact cryoconite hole size such as those in our study) is likely an important microbial adaptation to life on a glacier. Indeed, in a hostile environment where metabolism is slow due to cold conditions, rapid metabolic flexibility is likely not a viable option to survive and thrive. Overall, our functional data indicates that the core microbial communities in the surveyed cryoconite holes were relatively resilient to changes in environmental conditions (such as light and temperature) that affected their habitat during our sampling period. This result on functional and metabolic profiles builds on previous data for the GrIS highlighting the stability of community composition (Musilova et al. 2015, Stibal et al. 2015), allowing us to conclude that core metabolism, in particular photosynthesis, is also stable during the melt season. The high abundance in photosynthesis transcripts in all sites and time points, compared to transcripts linked to respiration or other nutrient cycling (such as nitrogen or sulfur), corroborates our knowledge that cryoconite hole communities are important carbon fixers and producers of organic carbon on the GrIS (Musilova et al. 2017). This result also corroborates previous data from Svalbard cryoconite metabolomes that highlighted that photosynthesis-related metabolites were not spatially variable across an ice cap (Gokul et al. 2023), implying crucial importance of photosynthesis for community function as well as photosynthetic resilience to changes in weather (in particular solar radiation). Metatranscriptomics data (and other meta-omics data) can be a predictor of the driving metabolisms and associated nutrient cycling in an ecosystem (for example Griffin et al. 2025). However, how important carbon fixation is compared to other cycling of other nutrients in terms of biogeochemical cycling rates will need to be further investigated empirically by directly measuring nutrient cycling rates.

## Conclusion

Our study allows us to answer key ecological questions on microbial dynamics on the GrIS. We show that, while relatively similar, cryoconite hole community compositions are nevertheless, spatially distinct. Our results show stable and active cryoconite hole community composition over time, with variations in active community function which were small and transient and mostly linked to the transcription of glacier ice algae stress-related genes. Invariably, photosynthesis was the dominant function in the surveyed cryoconite hole microbiomes, and this was dominated by Cyanobacteria and to a lesser extent Chlorophyta taxa. Overall, our data highlights that the core microbiomes of developed GrIS cryoconite hole are stable and resilient, ultimately allowing them to ensure essential ecosystem functions, such as carbon and nitrogen cycling, under changing environmental conditions

## Supplementary Material

fiag074_Supplemental_Files

## Data Availability

The data presented in this manuscript is available on NCBI: https://www.ncbi.nlm.nih.gov, accession number PRJNA1160058.

## References

[bib1] Adrian Alexa JR . topGO. Bioconductor, 2017. 10.18129/B9.BIOC.TOPGO (12 March 2026, date last accessed).

[bib2] Anesio AM, Hodson AJ, Fritz A et al. High microbial activity on glaciers: importance to the global carbon cycle. Global Change Biol. 2009;15:955–60. 10.1111/j.1365-2486.2008.01758.x

[bib3] Anesio AM, Lutz S, Chrismas NAM et al. The microbiome of glaciers and ice sheets. NPJ Biofilms Microbiomes. 2017;3:10. 10.1038/s41522-017-0019-0 28649411 PMC5460203

[bib4] Antony R, Mongad D, Sanyal A et al. Holed up, but thriving: impact of multitrophic cryoconite communities on glacier elemental cycles. Sci Total Environ. 2024;933:173187. 10.1016/j.scitotenv.2024.173187 38750762

[bib5] Bagshaw EA, Tranter M, Fountain AG et al. Biogeochemical evolution of cryoconite holes on Canada Glacier, Taylor Valley, Antarctica. J Geophys Res. 2007;112:2007JG000442. 10.1029/2007JG000442

[bib6] Bourquin M, Busi SB, Fodelianakis S et al. The microbiome of cryospheric ecosystems. Nat Commun. 2022;13:3087. 10.1038/s41467-022-30816-4 35655063 PMC9163120

[bib7] Buchfink B, Xie C, Huson DH. Fast and sensitive protein alignment using DIAMOND. Nat Methods. 2015;12:59–60. 10.1038/nmeth.3176 25402007

[bib8] Cameron KA, Hodson AJ, Osborn AM. Structure and diversity of bacterial, eukaryotic and archaeal communities in glacial cryoconite holes from the Arctic and the Antarctic. FEMS Microbiol Ecol. 2012;82:254–67. 10.1111/j.1574-6941.2011.01277.x 22168226

[bib9] Cameron KA, Stibal M, Zarsky JD et al. Supraglacial bacterial community structures vary across the Greenland ice sheet. FEMS Microbiol Ecol. 2016;92:fiv164. 10.1093/femsec/fiv164 26691594

[bib10] Capella-Gutiérrez S, Silla-Martínez JM, Gabaldón T. trimAl: a tool for automated alignment trimming in large-scale phylogenetic analyses. Bioinformatics. 2009;25:1972–3. 10.1093/bioinformatics/btp348 19505945 PMC2712344

[bib11] Chandler DM, Alcock JD, Wadham JL et al. Seasonal changes of ice surface characteristics and productivity in the ablation zone of the Greenland Ice Sheet. Cryosphere. 2015;9:487–504. 10.5194/tc-9-487-2015

[bib12] Chen Y, Liu Y, Ji M et al. Metabolic diversity and adaptation of carbon-fixing microorganisms in extreme glacial cryoconite. ISME Commun. 2025;5:ycaf056. 10.1093/ismeco/ycaf056 40259990 PMC12011081

[bib13] Chen Y, Liu Y, Liu K et al. Snowstorm enhanced the deterministic processes of the microbial community in cryoconite at Laohugou Glacier, Tibetan Plateau. Front Microbiol. 2022;12:784273. 10.3389/fmicb.2021.784273 35154026 PMC8829297

[bib14] Chrismas NAM, Barker G, Anesio AM et al. Genomic mechanisms for cold tolerance and production of exopolysaccharides in the Arctic cyanobacterium Phormidesmis priestleyi BC1401. Bmc Genom [Electron Resour]. 2016;17:533. 10.1186/s12864-016-2846-4 PMC4971617 27485510

[bib15] Cook J, Edwards A, Takeuchi N et al. Cryoconite: the dark biological secret of the cryosphere. Prog Phys Geogr Earth Environ. 2016;40:66–111. 10.1177/0309133315616574

[bib16] Cook JM, Edwards A, Bulling M et al. Metabolome-mediated biocryomorphic evolution promotes carbon fixation in greenlandic cryoconite holes. Environ Microbiol. 2016;18:4674–86. 10.1111/1462-2920.13349 27113725

[bib17] Danecek P, Bonfield JK, Liddle J et al. Twelve years of SAMtools and BCFtools. GigaScience. 2021;10:giab008. 10.1093/gigascience/giab008 PMC7931819 33590861

[bib18] Darcy JL, Gendron EMS, Sommers P et al. Island biogeography of cryoconite hole bacteria in Antarctica’s Taylor Valley and around the world. Front Ecol Evol. 2018;6:180. 10.3389/fevo.2018.00180

[bib19] Dixon P . VEGAN, a package of R functions for community ecology. J Vegetation Sci. 2003;14:927–30. 10.1111/j.1654-1103.2003.tb02228.x

[bib20] Edwards A, Anesio AM, Rassner SM et al. Possible interactions between bacterial diversity, microbial activity and supraglacial hydrology of cryoconite holes in Svalbard. ISME J. 2011;5:150–60. 10.1038/ismej.2010.100 20664552 PMC3105670

[bib21] Edwards A, Douglas B, Anesio AM et al. A distinctive fungal community inhabiting cryoconite holes on glaciers in Svalbard. Fungal Ecol. 2013;6:168–76. 10.1016/j.funeco.2012.11.001

[bib22] Edwards A, Mur LAJ, Girdwood SE et al. Coupled cryoconite ecosystem structure-function relationships are revealed by comparing bacterial communities in alpine and Arctic glaciers. FEMS Microbiol Ecol. 2014;89:222–37. 10.1111/1574-6941.12283 24433483

[bib23] Ezzedine JA, Uwizeye C, Si Larbi G et al. Adaptive traits of cysts of the snow alga Sanguina nivaloides unveiled by 3D subcellular imaging. Nat Commun. 2023;14:7500. 10.1038/s41467-023-43030-7 37980360 PMC10657455

[bib24] Fausto RS, Van As D, Mankoff KD et al. Programme for monitoring of the Greenland Ice Sheet (PROMICE) automatic weather station data. Earth Syst Sci Data. 2021;13:3819–45. 10.5194/essd-13-3819-2021

[bib25] Feord HK, Keuschnig C, Trivedi CB et al. Linking extreme light availability to cellular function in algae-dominated communities on the Greenland Ice Sheet. FEMS Microbiol Ecol. 2025;101:fiaf095. 10.1093/femsec/fiaf095 41014222 PMC12501423

[bib26] Feord HK, Trautwein-Schult A, Keuschnig C et al. Algae-dominated metaproteomes uncover cellular adaptations to life on the Greenland Ice Sheet. npj Biofilms Microbiomes. 2025;11:181. 10.1038/s41522-025-00770-2 40925914 PMC12420790

[bib27] Franzetti A, Navarra F, Tagliaferri I et al. Temporal variability of bacterial communities in cryoconite on an alpine glacier. Environ Microbiol Rep. 2017;9:71–8. 10.1111/1758-2229.12499 27897429

[bib28] Ge Q, Chen Z, Xu Y et al. Biogeography of cryoconite bacterial communities across continents. Microorganisms. 2026;14:162. 10.3390/microorganisms14010162 41597681 PMC12844174

[bib29] Gokul JK, Hodson AJ, Saetnan ER et al. Taxon interactions control the distributions of cryoconite bacteria colonizing a High Arctic ice cap. Mol Ecol. 2016;25:3752–67. 10.1111/mec.13715 27261672

[bib30] Gokul JK, Mur LAJ, Hodson AJ et al. Icescape-scale metabolomics reveals cyanobacterial and topographic control of the core metabolism of the cryoconite ecosystem of an Arctic ice cap. Environ Microbiol. 2023;25:2549–63. 10.1111/1462-2920.16485 37621052

[bib31] Griffin NA, Kim B, Hardison AK et al. Quantitative metatranscriptomics and biogeochemical rate measurements reveal microbial pathways driving carbon and nitrogen cycles in an Arctic coastal lagoon. Limnol Oceanogr. 2025;70:3725–40. 10.1002/lno.70257

[bib32] Gurevich A, Saveliev V, Vyahhi N et al. QUAST: quality assessment tool for genome assemblies. Bioinformatics. 2013;29:1072–5. 10.1093/bioinformatics/btt086 23422339 PMC3624806

[bib33] Hamilton TL, Havig JR. Addition of dissolved inorganic carbon stimulates snow algae primary productivity on glacially eroded carbonate bedrock in the Medicine Bow Mountains, WY, USA. FEMS Microbiol Ecol. 2023;99:fiad056. 10.1093/femsec/fiad056 37222475 PMC10289208

[bib34] Huson DH, Beier S, Flade I et al. MEGAN community edition—interactive exploration and analysis of large-scale microbiome sequencing data. PLoS Comput Biol. 2016;12:e1004957. 10.1371/journal.pcbi.1004957 27327495 PMC4915700

[bib35] Jaarsma AH, Sipes K, Zervas A et al. Exploring microbial diversity in Greenland Ice Sheet supraglacial habitats through culturing-dependent and -independent approaches. FEMS Microbiol Ecol. 2023;99:fiad119. 10.1093/femsec/fiad119 37791411 PMC10580271

[bib36] Jaarsma AH, Sipes K, Zervas A et al. The encoded and expressed biosynthetic potential of Greenland Ice Sheet microbes. Front Microbiol. 2025;16:1620548. 10.3389/fmicb.2025.1620548 40822389 PMC12350317

[bib37] Jaroměřská TN, Ambrosini R, Richter D et al. Insights into cryoconite community dynamics on the Alpine Glacier throughout the ablation season. Ecol Evol. 2025;15:e71064. 10.1002/ece3.71064 40130008 PMC11932729

[bib38] Jiménez FC . AU-ENVS-Bioinformatics/TotalRNA-Snakemake: v1.1.1, version v.1.0.1. Zenodo, 2023. 10.5281/ZENODO.7656004 (17 December 2025, date last accessed).

[bib39] Jones P, Binns D, Chang HY et al. InterProScan 5: genome-scale protein function classification. Bioinformatics. 2014;30:1236–40. 10.1093/bioinformatics/btu031 24451626 PMC3998142

[bib40] Kanehisa M, Sato Y, Morishima K. BlastKOALA and GhostKOALA: KEGG tools for functional characterization of genome and metagenome sequences. J Mol Biol. 2016;428:726–31. 10.1016/j.jmb.2015.11.006 26585406

[bib41] Kobayashi K, Takeuchi N, Kagami M. High prevalence of parasitic chytrids infection of glacier algae in cryoconite holes in Alaska. Sci Rep. 2023;13:3973. 10.1038/s41598-023-30721-w 36894609 PMC9998860

[bib42] Kopylova E, Noé L, Touzet H. SortMeRNA: fast and accurate filtering of ribosomal RNAs in metatranscriptomic data. Bioinformatics. 2012;28:3211–7. 10.1093/bioinformatics/bts611 23071270

[bib43] Kumar L, Futschik ME. Mfuzz: a software package for soft clustering of microarray data. Bioinformation. 2007;2:5–7. 10.6026/97320630002005 18084642 PMC2139991

[bib44] Langford H, Hodson A, Banwart S et al. The microstructure and biogeochemistry of Arctic cryoconite granules. Ann Glaciol. 2010;51:87–94. 10.3189/172756411795932083

[bib45] Lanzén A, Jørgensen SL, Huson DH et al. CREST—classification resources for environmental sequence tags. PLoS One. 2012;7:e49334. 10.1371/journal.pone.0049334 23145153 PMC3493522

[bib46] Letunic I, Bork P. Interactive Tree of Life (iTOL) v6: recent updates to the phylogenetic tree display and annotation tool. Nucleic Acids Res. 2024;52:W78–82. 10.1093/nar/gkae268 38613393 PMC11223838

[bib47] Li H, Durbin R. Fast and accurate short read alignment with Burrows–Wheeler transform. Bioinformatics. 2009;25:1754–60. 10.1093/bioinformatics/btp324 19451168 PMC2705234

[bib48] Liu Y, Vick-Majors TJ, Priscu JC et al. Biogeography of cryoconite bacterial communities on glaciers of the Tibetan Plateau. FEMS Microbiol Ecol. 2017;93:fix072. 10.1093/femsec/fix072 28531262

[bib49] Love MI, Huber W, Anders S. Moderated estimation of fold change and dispersion for RNA-seq data with DESeq2. Genome Biol. 2014;15:550. 10.1186/s13059-014-0550-8 25516281 PMC4302049

[bib50] Lutz S, Ziolkowski LA, Benning LG. The biodiversity and geochemistry of cryoconite holes in Queen Maud Land, East Antarctica. Microorganisms. 2019;7:160. 10.3390/microorganisms7060160 31159414 PMC6616603

[bib51] McMurdie PJ, Holmes S. phyloseq: an R package for reproducible interactive analysis and graphics of microbiome census data. PLoS One. 2013;8:e61217. 10.1371/journal.pone.0061217 23630581 PMC3632530

[bib52] Millar JL, Bagshaw EA, Edwards A et al. Polar cryoconite associated microbiota is dominated by hemispheric specialist genera. Front Microbiol. 2021;12:738451. 10.3389/fmicb.2021.738451 34899626 PMC8660574

[bib53] Minh BQ, Schmidt HA, Chernomor O et al. IQ-TREE 2: new models and efficient methods for phylogenetic inference in the genomic era. Mol Biol Evol. 2020;37:1530–4. 10.1093/molbev/msaa015 32011700 PMC7182206

[bib54] Murakami T, Takeuchi N, Mori H et al. Metagenomics reveals global-scale contrasts in nitrogen cycling and cyanobacterial light-harvesting mechanisms in glacier cryoconite. Microbiome. 2022;10:50. 10.1186/s40168-022-01238-7 35317857 PMC8941735

[bib55] Musilova M, Tranter M, Bennett SA et al. Stable microbial community composition on the Greenland Ice Sheet. Front Microbiol. 2015;6. 10.3389/fmicb.2015.00193 PMC4367435 25852658

[bib56] Musilova M, Tranter M, Wadham J et al. Microbially driven export of labile organic carbon from the Greenland ice sheet. Nature Geosci. 2017;10:360–5. 10.1038/ngeo2920

[bib57] Nordenskiöld A . Cryoconite found 1870, July 19th-25th, on the inland ice, east of Auleitsivik Fjord, Disco Bay, Greenland. Geol Mag. 1875;2:157–62.

[bib58] Perini L, Sipes K, Zervas A et al. Giant viral signatures on the Greenland Ice Sheet. Microbiome. 2024;12:art.1. 10.1186/s40168-024-01796-y PMC11100222 38760842

[bib59] Perini L, Zervas A, Feld L et al. The diversity and abundance of chytrids on the Greenland Ice Sheet. Sci Rep. 2026;16:11175. 10.1038/s41598-026-41468-5 41748675 PMC13046730

[bib60] Pessa JC, Joutsen J, Sistonen L. Transcriptional reprogramming at the intersection of the heat shock response and proteostasis. Mol Cell. 2024;84:80–93. 10.1016/j.molcel.2023.11.024 38103561

[bib61] Pittino F, Maglio M, Gandolfi I et al. Bacterial communities of cryoconite holes of a temperate alpine glacier show both seasonal trends and year-to-year variability. Ann Glaciol. 2018;59:1–9. 10.1017/aog.2018.16

[bib62] Poniecka EA, Bagshaw EA, Tranter M et al. Rapid development of anoxic niches in supraglacial ecosystems. Arct Antarct Alp Res. 2018;50:S100015. 10.1080/15230430.2017.1420859

[bib63] Pratx L, Crawford T, Bäurle I. Mechanisms of heat stress-induced transcriptional memory. Curr Opin Plant Biol. 2024;81:102590. 10.1016/j.pbi.2024.102590 38968911

[bib64] R Core Team . R: a Language and Environment for Statistical Computing. 2023. https://www.r-project.org

[bib65] Rozwalak P, Podkowa P, Buda J et al. Cryoconite—from minerals and organic matter to bioengineered sediments on glacier’s surfaces. Sci Total Environ. 2022;807:150874. 10.1016/j.scitotenv.2021.150874 34627905

[bib66] Ruvindy R, Barua A, Bolch CJS et al. Genomic copy number variability at the genus, species and population levels impacts in situ ecological analyses of dinoflagellates and harmful algal blooms. ISME Commun. 2023;3:70. 10.1038/s43705-023-00274-0 37422553 PMC10329664

[bib67] Sanyal A, Antony R, Samui G et al. Microbial communities and their potential for degradation of dissolved organic carbon in cryoconite hole environments of Himalaya and Antarctica. Microbiol Res. 2018;208:32–42. 10.1016/j.micres.2018.01.004 29551210

[bib68] Sanyal A, Antony R, Samui G et al. Autotrophy to heterotrophy: shift in bacterial functions during the melt season in Antarctic Cryoconite Holes. J Microbiol. 2024;62:591–609. 10.1007/s12275-024-00140-1 38814540

[bib69] Scheel M, Zervas A, Rijkers R et al. Abrupt permafrost thaw triggers activity of copiotrophs and microbiome predators. FEMS Microbiol Ecol. 2023;99:fiad123. 10.1093/femsec/fiad123 37796894 PMC10599396

[bib70] Schneider CA, Rasband WS, Eliceiri KW. NIH Image to ImageJ: 25 years of image analysis. Nat Methods. 2012;9:671–5. 10.1038/nmeth.2089 22930834 PMC5554542

[bib71] Segawa T, Ishii S, Ohte N et al. The nitrogen cycle in cryoconites: naturally occurring nitrification-denitrification granules on a glacier. Environ Microbiol. 2014;16:3250–62. 10.1111/1462-2920.12543 24946985

[bib72] Segawa T, Takeuchi N, Mori H et al. Redox stratification within cryoconite granules influences the nitrogen cycle on glaciers. FEMS Microbiol Ecol. 2020;96:fiaa199. 10.1093/femsec/fiaa199 32990745

[bib73] Segawa T, Yonezawa T, Edwards A et al. Biogeography of cryoconite forming cyanobacteria on polar and Asian glaciers. J Biogeogr. 2017;44:2849–61. 10.1111/jbi.13089

[bib74] Sievers F, Wilm A, Dineen D et al. Fast, scalable generation of high-quality protein multiple sequence alignments using Clustal Omega. Mol Syst Biol. 2011;7:539. 10.1038/msb.2011.75 21988835 PMC3261699

[bib75] Stibal M, Lawson EC, Lis GP et al. Organic matter content and quality in supraglacial debris across the ablation zone of the Greenland ice sheet. Ann Glaciol. 2010;51:1–8. 10.3189/172756411795931958

[bib76] Stibal M, Schostag M, Cameron KA et al. Different bulk and active bacterial communities in cryoconite from the margin and interior of the g reenland ice sheet. Environ Microbiol Rep. 2015;7:293–300. 10.1111/1758-2229.12246 25405749

[bib77] Strunecký O, Ivanova AP, Mareš J. An updated classification of cyanobacterial orders and families based on phylogenomic and polyphasic analysis. J Phycol. 2023;59:12–51. 10.1111/jpy.13304 36443823

[bib78] Takeuchi N, Murakami T, Ishiwatari K et al. Morphology shapes microbial ecosystems and carbon cycling within cryoconite holes on a Greenland outlet glacier. Commun Earth Environ. 2025;7:16. 10.1038/s43247-025-03045-y

[bib79] Takeuchi N, Nishiyama H, Li Z. Structure and formation process of cryoconite granules on Ürümqi glacier No. 1, Tien Shan, China. Ann Glaciol. 2010;51:9–14. 10.3189/172756411795932010

[bib80] Takeuchi N, Sakaki R, Uetake J et al. Temporal variations of cryoconite holes and cryoconite coverage on the ablation ice surface of Qaanaaq Glacier in northwest Greenland. Ann Glaciol. 2018;59:21–30. 10.1017/aog.2018.19

[bib81] Trivedi CB, Keuschnig C, Larose C et al. DNA/RNA preservation in glacial snow and ice samples. Front Microbiol. 2022;13:894893. 10.3389/fmicb.2022.894893 35677909 PMC9168539

[bib82] Uetake J, Tanaka S, Segawa T et al. Microbial community variation in cryoconite granules on Qaanaaq Glacier, NW Greenland. FEMS Microbiol Ecol. 2016;92:fiw127. 10.1093/femsec/fiw127 27306554

[bib83] Vonnahme TR, Devetter M, Žárský JD et al. Controls on microalgal community structures in cryoconite holes upon high-Arctic glaciers, Svalbard. Biogeosciences. 2016;13:659–74. 10.5194/bg-13-659-2016

[bib84] Wejnerowski Ł, Poniecka E, Buda J et al. Empirical testing of cryoconite granulation: role of cyanobacteria in the formation of key biogenic structure darkening glaciers in polar regions. J Phycol. 2023;59:939–49. 10.1111/jpy.13372 37572353

[bib85] Xue Y, Lanzén A, Jonassen I. Reconstructing ribosomal genes from large scale total RNA meta-transcriptomic data. Bioinformatics. 2020;36:3365–71. 10.1093/bioinformatics/btaa177 32167532 PMC7267836

[bib86] Zawierucha K, Buda J, Nawrot A. Extreme weather event results in the removal of invertebrates from cryoconite holes on an Arctic valley glacier (Longyearbreen, Svalbard). Ecol Res. 2019;34:370–9. 10.1111/1440-1703.1276

[bib87] Zawierucha K, Porazinska DL, Ficetola GF et al. A hole in the nematosphere: tardigrades and rotifers dominate the cryoconite hole environment, whereas nematodes are missing. J Zool. 2021;313:18–36. 10.1111/jzo.12832

[bib88] Zhang Z, Liu Y, Zhao W et al. Radiation impacts gene redundancy and biofilm regulation of cryoconite microbiomes in Northern Hemisphere glaciers. Microbiome. 2023;11:228. 10.1186/s40168-023-01621-y 37848997 PMC10583317

[bib89] Zhu X, Liu K, Liu Y et al. Cyanobacteria sustain microbial diversity and community stability in Tibetan glacial cryoconites. Environ Microb. 2025;20:152. 10.1186/s40793-025-00817-z PMC12709721 41408587

[bib90] Zippenfenig P . Open-Meteo.Com Weather API, version 1.4.0. Zenodo, 2024. 10.5281/ZENODO.7970649 (30 June 2026, date last accessed).

