# Supplementary material for: Highly stable active core microbiomes in Greenland cryoconite holes during the bare ice period
Source: FEMS Microbiol Ecol. 2026 Jul 8;102(8):fiag074. doi: 10.1093/femsec/fiag074 (PMC13390577; doi:10.1093/femsec/fiag074)
Supplement: fiag074_Supplemental_Files [file fiag074_supplemental_files.zip › 2606_ILU_cc_suppfigs_1.docx]

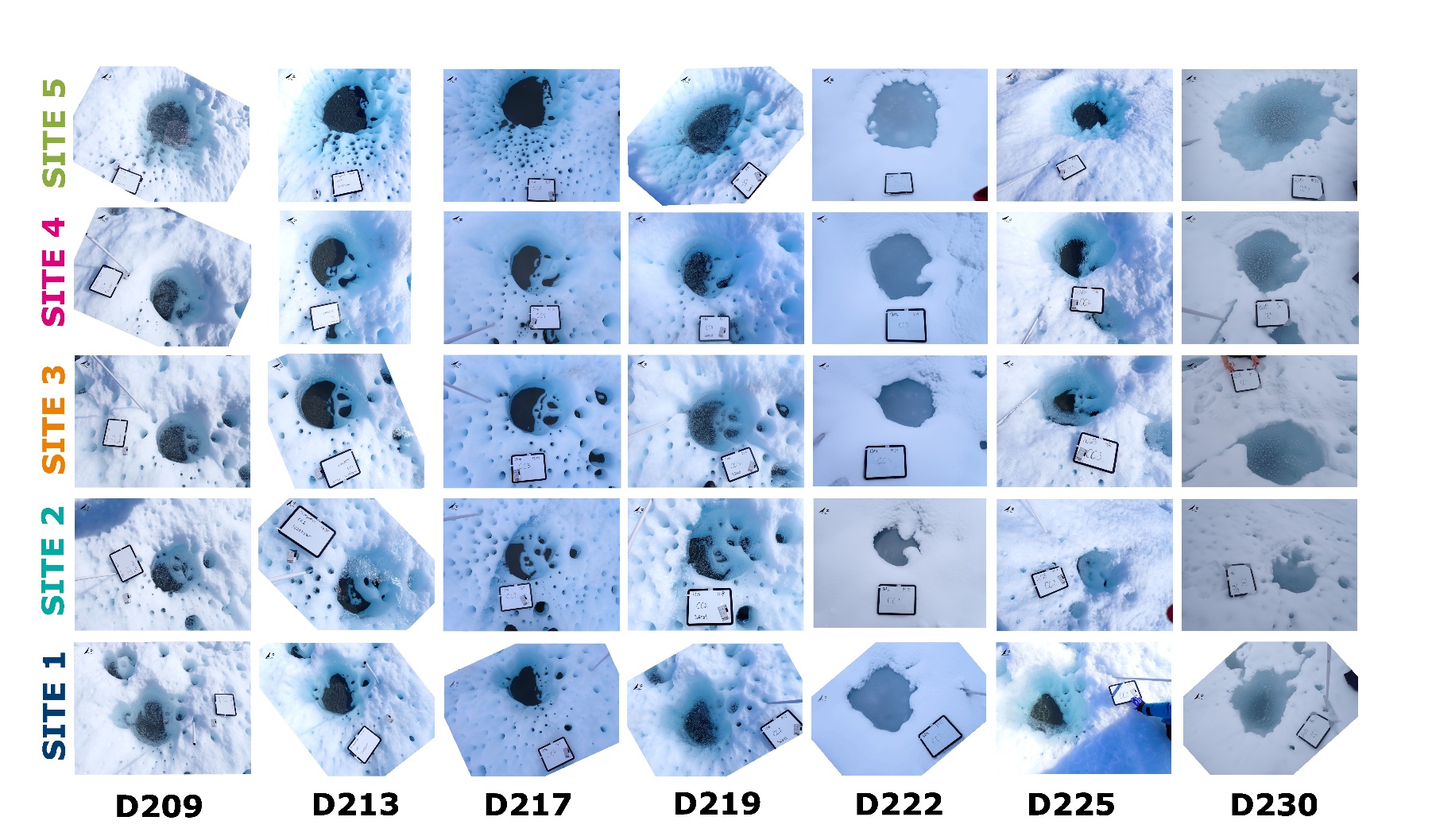


SFigure 1: Cryoconite holes sampled during the three-week sampling campaign on the Greenland Ice Sheet. Pictures of the five cryoconite holes (Sites 1-5) are shown on the seven sampling days (D209-230) with the white board in each picture as scale (~30 cm length). Certain pictures have been rotated in order to retain the same position in all pictures for each site.


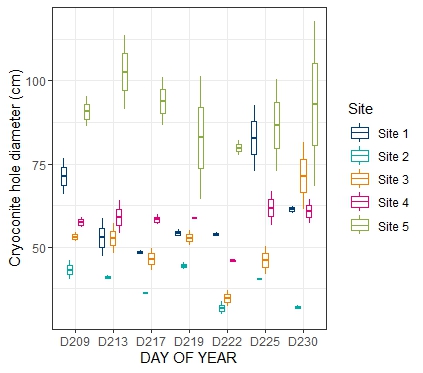


SFigure 2: Variation of cryoconite hole diameter over space and time. Cryoconite hole diameter was measured in duplicate using perpendicular measurements from cryoconite hold pictures available in Supplementary Figure 1. For each site/time point, the diameter was measured to include the extremities of the water surface, rather than simply measuring the physical ice boundaries of deepest part of the cryoconite hole. For cryoconite hole measurements after snow events, the diameter was measured between the snow edges.


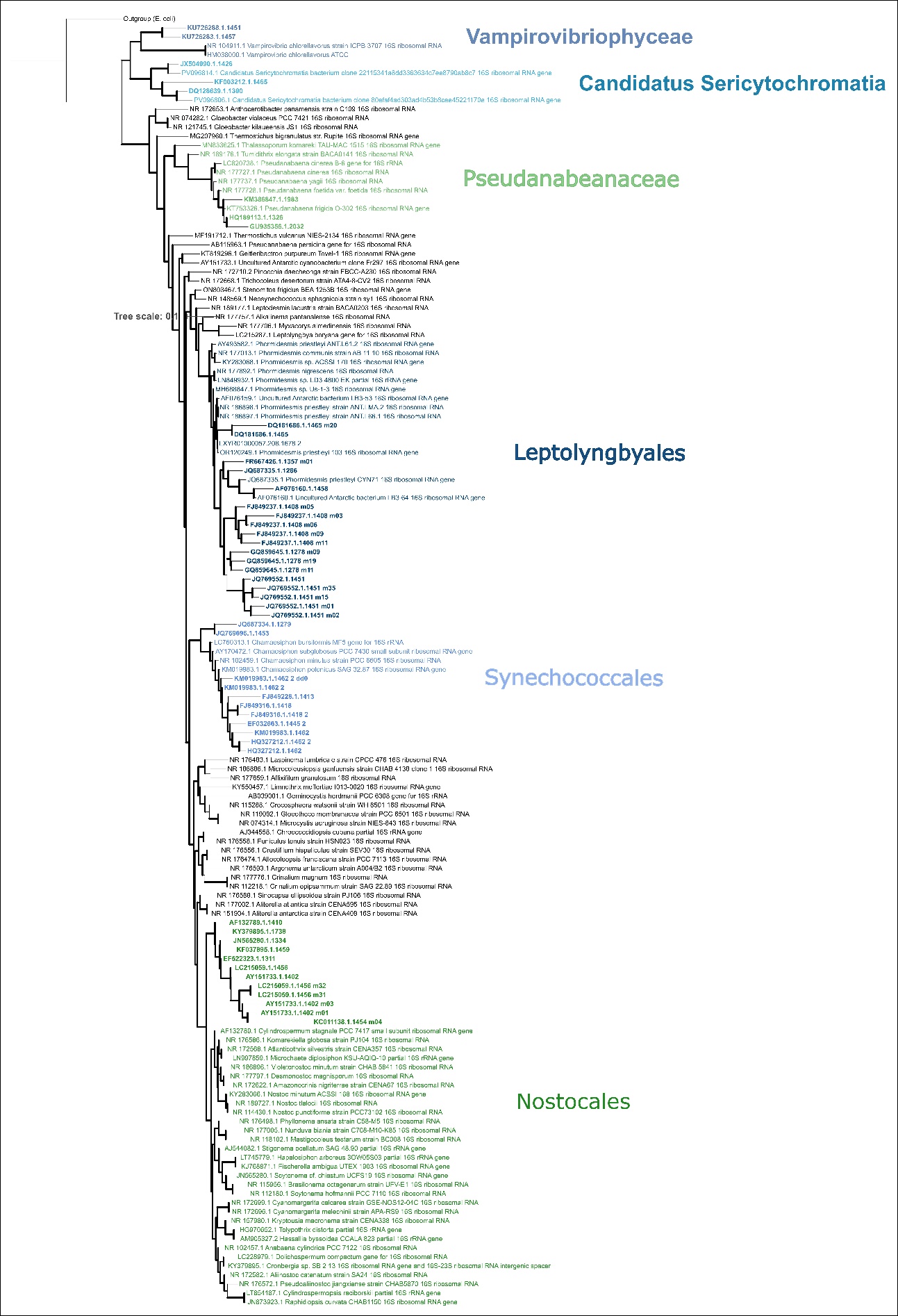


**SFigure 3: Maximum likelihood tree (1000 bootstraps) showing the phylogenetic placement of cyanobacterial rRNA SSUs from our study (in bold) compared to publicly available sequences.** Colour coding matches the order/family categories of Figure 5.


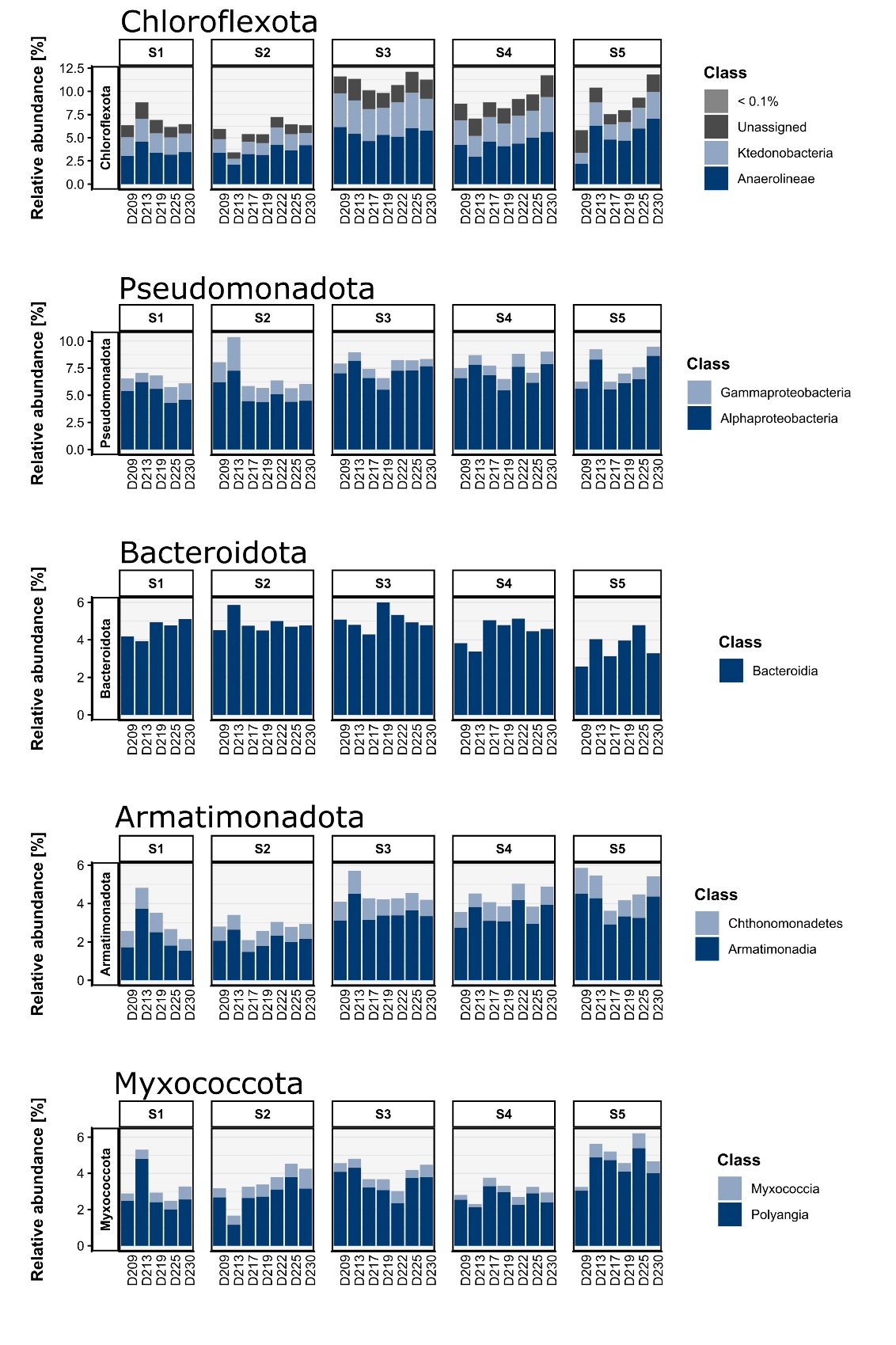


**SFigure 4: Relative abundance of different active bacterial cryoconite hole community members based on taxonomic annotation of rRNA SSUs at class level.** Relative abundance is plotted for each cryoconite hole (Sites 1-5) across the different sampling days (D209-230).


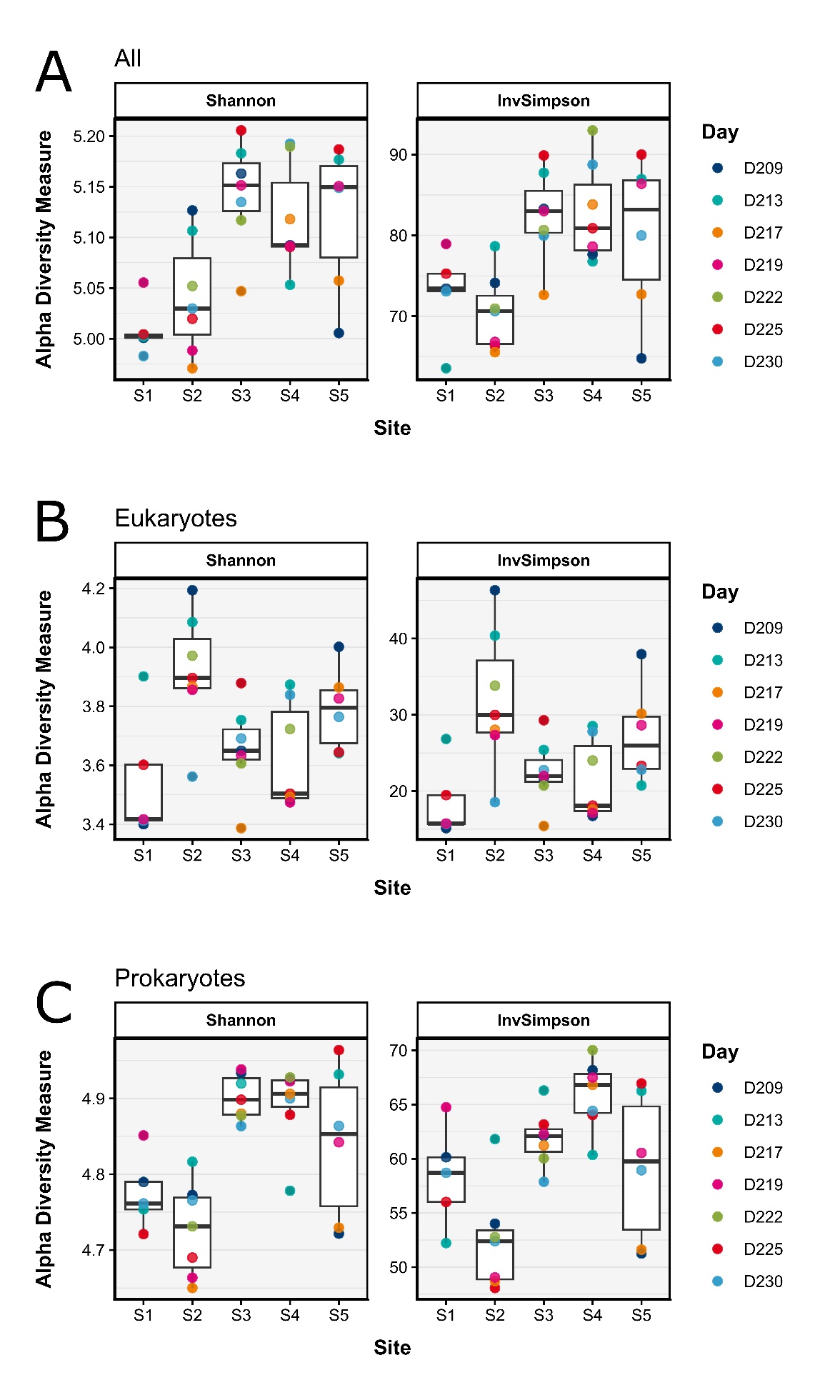
**SFigure 5: Alpha diversity indices for (A) total and (B) eukaryotic and (C) prokaryotic fractions.** The measures shown are Shannon and Inverse Simpson.


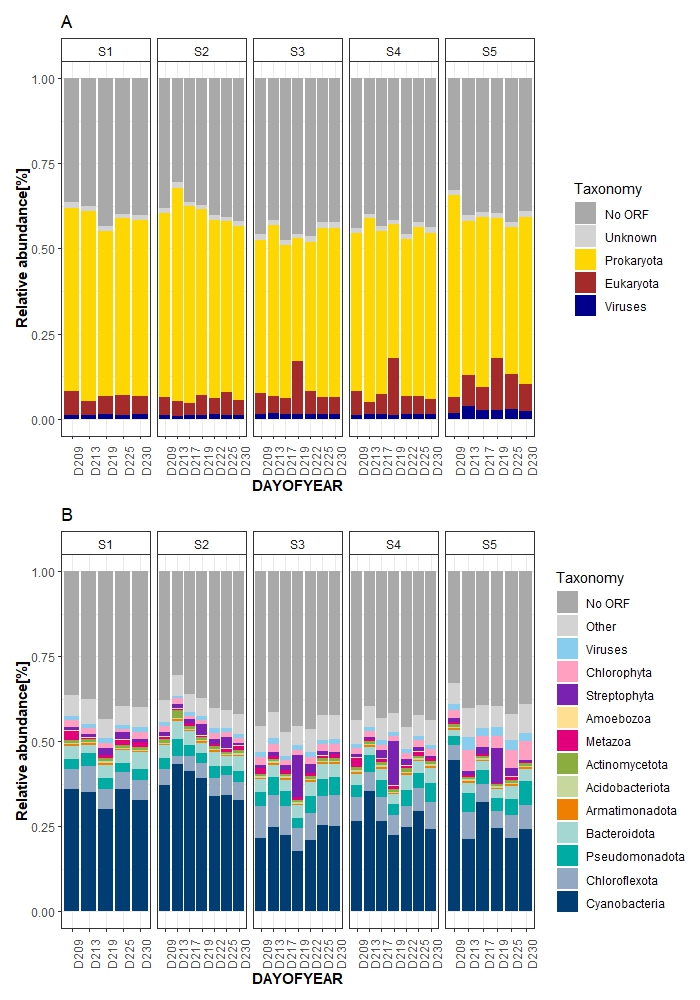


**SFigure 6: Relative abundance of active cryoconite hole community members based on taxonomic annotation of mRNA using MEGAN.** Relative abundance is plotted for each cryoconite hole (Sites 1-5) across the different sampling days (D209-230). Plots for kingdom-level annotations (**A**) are shown alongside higher resolution (mostly phyla) annotations (**B**).


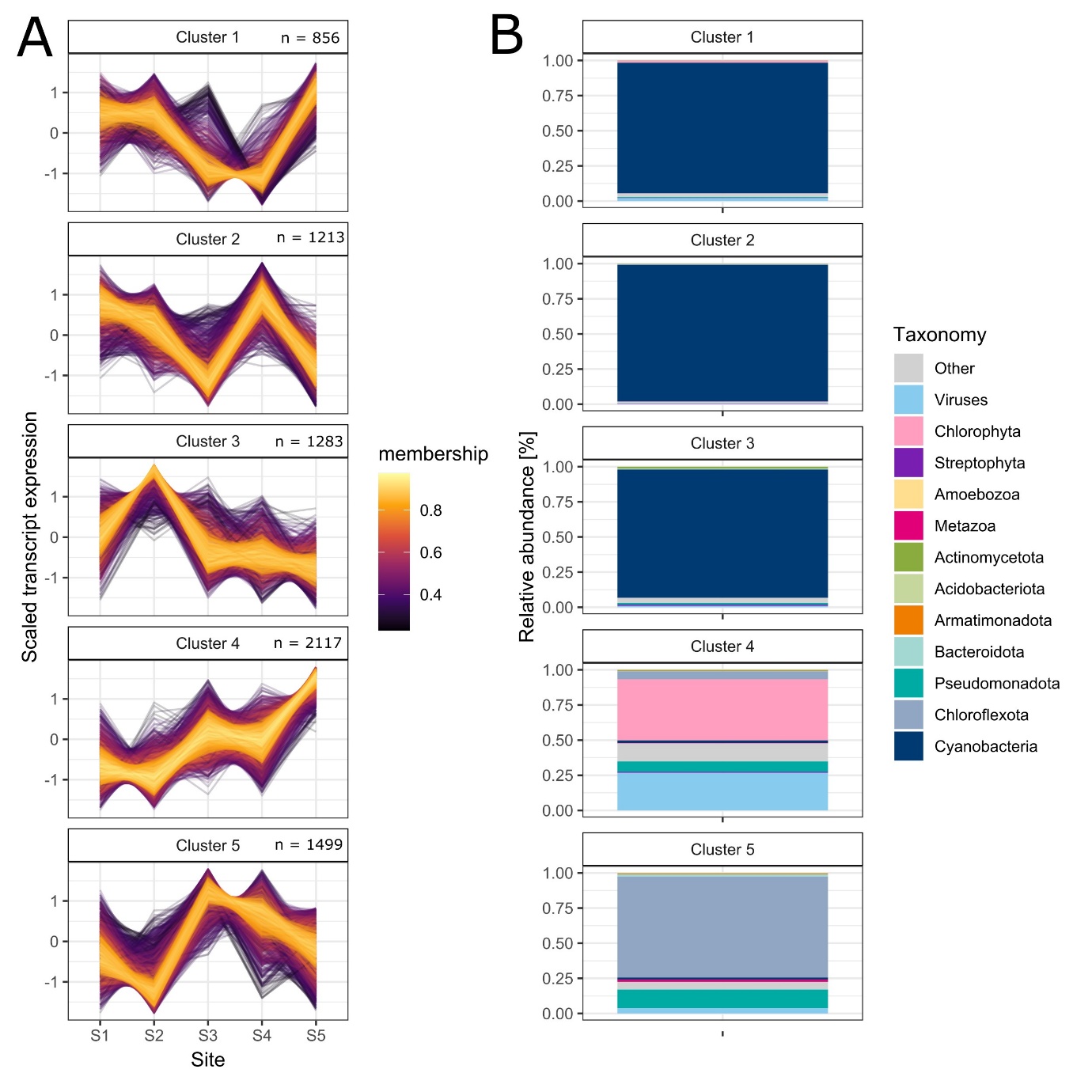


SFigure 7: Expression clusters of spatially varying transcripts. The expression trends of the five clusters (A) are plotted by using scaled transcript expression and color coded by cluster membership, with light colors indicating high cluster membership and darker colors low cluster membership, and are separated by site. The (B) taxonomy of the individual cluster transcripts is plotted as the mean relative abundance of these transcript counts for the sample days with the highest expression (site 5 for cluster 1, site 2 for cluster 2, site 2 for cluster 3, site 5 for cluster 4, site 3 for cluster 5).


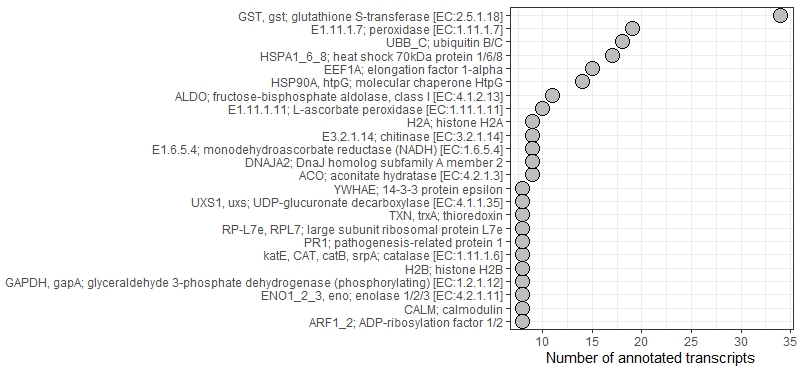


**SFigure 8: Enriched KEGG Orthology (GO) terms in cluster 1.** Transcripts from cluster 1 were annotated with GhostKOALA (see methods) and the number of transcripts for each annotation was summed.


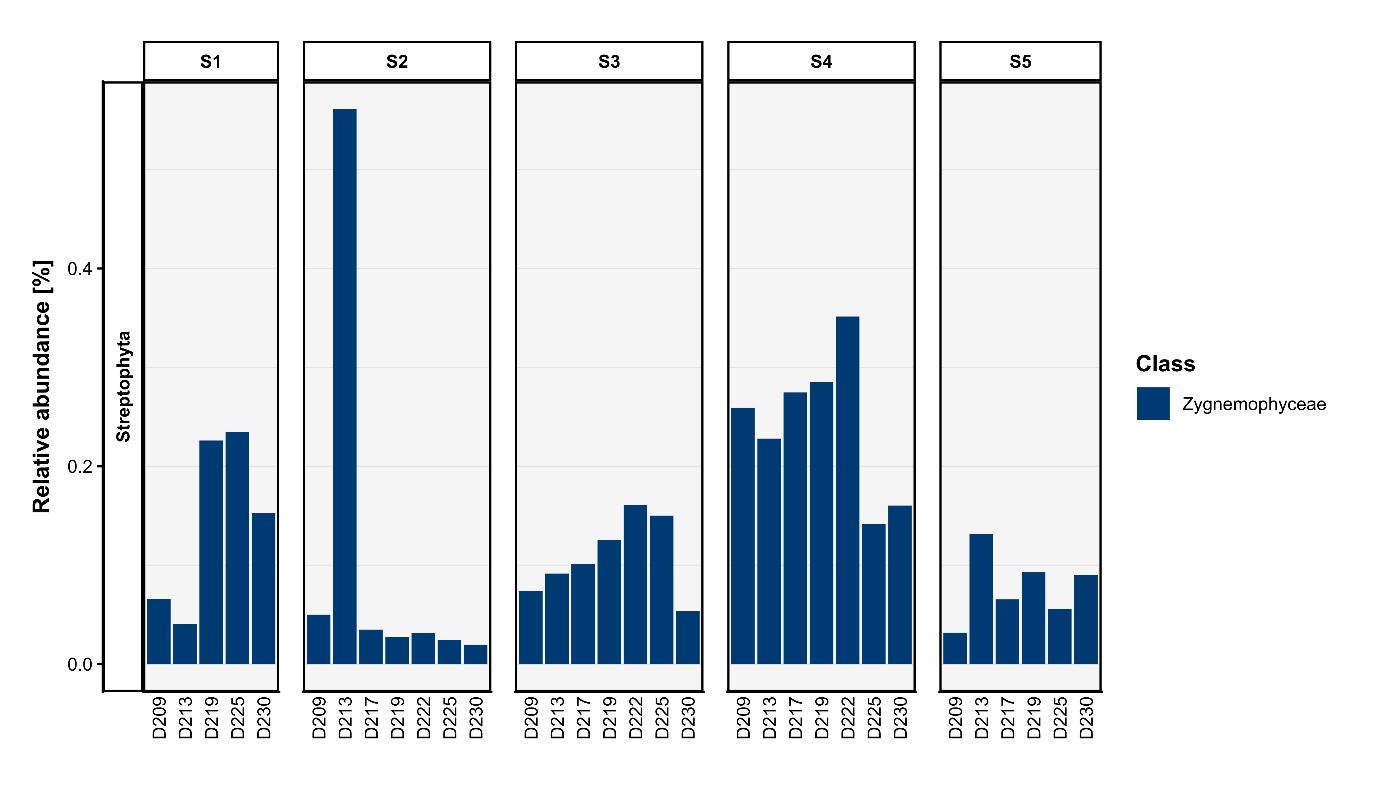


**SFigure 9: Streptophyte rRNA SSUs in the surveyed cryoconite hole communities over time.** Relative abundance is plotted for each cryoconite hole (site 1-5) across the different sampling points (D209-230).
